# Development of protein biomarkers in cerebrospinal fluid for secondary progressive multiple sclerosis using selected reaction monitoring mass spectrometry (SRM-MS)

**DOI:** 10.1186/1559-0275-9-9

**Published:** 2012-07-30

**Authors:** Yan Jia, Tianxia Wu, Christine A Jelinek, Bibiana Bielekova, Linda Chang, Scott Newsome, Sharmilee Gnanapavan, Gavin Giovannoni, Dawn Chen, Peter A Calabresi, Avindra Nath, Robert J Cotter

**Affiliations:** 1 Department of Pharmacology and Molecular Sciences, School of Medicine, Johns Hopkins University, Baltimore, MD, 21205, USA; 2 National Institute of Neurological Diseases and Stroke, National Institutes of Health, Bethesda, MD, USA; 3 Department of Internal Medicine, University of Hawaii, Manoa, HI, USA; 4 Department of Neurology, School of Medicine, Johns Hopkins University, Baltimore, MD, 21205, USA; 5 Department of Neuroimmunology, Institute of Neurology, Queen Square, WC1N 3BG, UK; 6 Department of Neuroimmunology, Barts and The London School of Medicine and Dentistry, London, E1 2AT, UK

## Abstract

**Background:**

*Multiple sclerosis* (MS) is a chronic inflammatory disorder of the central nervous system (CNS). It involves damage to the myelin sheath surrounding axons and to the axons themselves. MS most often presents with a series of relapses and remissions but then evolves over a variable period of time into a slowly progressive form of neurological dysfunction termed secondary progressive MS (SPMS). The reasons for this change in clinical presentation are unclear. The absence of a diagnostic marker means that there is a lag time of several years before the diagnosis of SPMS can be established. At the same time, understanding the mechanisms that underlie SPMS is critical to the development of rational therapies for this untreatable stage of the disease.

**Results:**

Using high performance liquid chromatography-coupled mass spectrometry (HPLC); we have established a highly specific and sensitive *selected reaction monitoring* (SRM) assay. Our multiplexed SRM assay has facilitated the simultaneous detection of surrogate peptides originating from 26 proteins present in cerebrospinal fluid (CSF). Protein levels in CSF were generally ~200-fold lower than that in human sera. A limit of detection (LOD) was determined to be as low as one femtomol. We processed and analysed CSF samples from a total of 22 patients with SPMS, 7 patients with SPMS treated with lamotrigine, 12 patients with non-inflammatory neurological disorders (NIND) and 10 healthy controls (HC) for the levels of these 26 selected potential protein biomarkers. Our SRM data found one protein showing significant difference between SPMS and HC, three proteins differing between SPMS and NIND, two proteins between NIND and HC, and 11 protein biomarkers showing significant difference between a lamotrigine-treated and untreated SPMS group. Principal component analysis (PCA) revealed that these 26 proteins were correlated, and could be represented by four principal components. Overall, we established an efficient platform to develop and verify protein biomarkers in CSF, which can be easily adapted to other proteins of interest related to neurodegenerative diseases.

**Conclusions:**

A highly specific and sensitive multiplex SRM-MS assay was established for development and verification of CSF protein biomarkers in SPMS. Five proteins were found to be expressed significantly differently between the three cohorts, SPMS, NIND and HC and 11 proteins associated with lamotrigine treatment, which we expect will further our current understanding of SPMS disease pathology and/or therapeutic intervention.

## Background

Multiple sclerosis (MS) is an inflammatory demyelinating disease of the CNS. During MS, myelin sheaths surrounding axons and the axons themselves are damaged as a result of chronic inflammation. Such inflammatory damage creates characteristic focal plaques in the white matter of the brain and spinal cord [1,2]. Patients with MS initially present with a host of non-specific neurological symptoms and there is a high degree of variability in the early symptoms that patients experience. For almost all patients, however, early MS associated symptoms will occur as a series of relapses and remissions whereby a patient will remain almost entirely asymptomatic for an extended period of time between temporary symptom “relapses.” Over a variable period of time, MS will typically evolve from *relapsing-remitting multiple sclerosis* (RRMS) into a slowly progressive form of neurological dysfunction termed *secondary progressive multiple sclerosis* (SPMS) [3].

For a complex disease like MS, there is a good reason to believe that a change in the protein expression profile occurs long before clinical symptoms are established. Therefore, protein biomarkers differentially expressed in MS patients as compared to healthy individuals or patients suffering from other neurological disorders have great clinical potential - not only as early diagnostics, but as potential prognostic markers to be used for monitoring disease course and evaluating treatment efficacy [4-6]. Identification of such markers would also further our current understanding of MS disease pathology, pinpointing novel protein targets or signalling pathways for therapeutic intervention.

Mass spectrometry-based proteomic technologies have become preferred laboratory strategies for the discovery of diagnostic, prognostic, and therapeutic biomarkers [7,8]. Since the early 1990s, a large number of potential protein biomarkers have been discovered every year in labs across the country - in both the academic and the industry sectors. However, only a handful of these identified targets are selected for further clinical investigation, fewer still have been formally validated, and of the thousands of markers that were identified between 2000 and 2005, only five new protein markers were approved by the US Food and Drug Administration (FDA) for measurement in sera or plasma in that time span [9]. Indeed, since 1998, the introduction of new protein biomarkers approved by the US FDA has fallen to an average of one per year; and this trend continues to be true today despite intensified interest and investment from both academia and industry. While to some extent, these types of statistics reflect the inherent time-line needed to conduct discovery-based research, it has long been suggested that development of faster and more efficient biomarker verification strategies could greatly reduce the time and cost of biomarker-based diagnostic development and that the greatest bottleneck to the current biomarker pipeline occurs at the verification/validation stage [10], that is the stage at which purported biomarkers, obtained through any number of *discovery* strategies, are tested quantitatively on samples derived from a statistically significant number of subjects and controls.

There have been two commonly adopted strategies for obtaining these kinds of quantitative assays for protein biomarkers: the *enzyme-linked immunosorbent assay* (ELISA) utilizing antibodies to the proteins and mass spectrometry-based methods that target the predictable fragmentation of surrogate peptides. Immunoassays enjoy high specificity and high throughput; however, they depend critically on the availability of highly specific antibodies, the development of which is usually long and costly. In addition, there are technical limitations for multiplexing of immunoassays. In contrast, quantitative approaches using *selected reaction monitoring* (SRM) mass spectrometry, have recently drawn intense interest to protein biomarker verification/validation for advantages of enhanced sensitivity and specificity, ease of high throughput, and relatively low cost [8,9,11-15]. Using a tandem mass spectrometer in which the mass analysers act as two mass filters, one for the peptide molecular ion mass and the other for a known sequence fragment, SRM-MS provides a selectivity and specificity that in many cases enables one to simplify sample preparation by avoiding the initial immunoprecipitation or fractionation common in many other mass spectrometry-based strategies.

CSF has been a valuable diagnostic resource for neurodegenerative diseases because its composition directly reflects the metabolic process of the brain. Five proteins including interleukin (IL)-17 have been recently identified by Trojanowaski and colleagues as CSF biomarkers capable of differentiating between two forms of frontotemporal lobar degeneration [16]. Similarly, amyloid beta (1–42), total tau and phosphorylated tau have been established as biomarkers for diagnosis of Alzheimer’s Disease (AD). Mattsson and colleagues found that combining biomarkers into a panel leads to a better predictive value than using individual biomarker for the diagnosis of AD [17]. Interestingly, while the three AD biomarkers identified by Mattsson, et al. [17] had diagnostic value when patient CSF was interrogated, the proteins were either not detected in the peripheral blood or their measured concentration in plasma did not provide useful information for diagnosis. Given the results from the Mattsson study and how varied MS clinical presentation and disease progression can be across patients, it is highly unlikely that a single protein biomarker will be sufficient for a conclusive diagnosis of SPMS. It is equally unlikely that a single protein would define the pathophysiology of SPMS. It is far more probable that a biomarker dependent diagnosis will require a panel of disease indicators whereby different indicators will have greater or lesser diagnostic utility depending upon individual patients or stages of disease evolution. As such, our overall objective is to find a panel of CSF protein biomarkers that show differential expression profiles amongst SPMS patients, patients suffering from non-inflammatory neurological disorders, and disease-free controls. For the studies presented here, we also included subjects from the placebo-controlled clinical trial for lamotrigine as a putative neuroprotective therapy in secondary progressive MS from the UK. Though this clinical trial did not demonstrate a significant difference in clinical outcomes between patients taking lamotrigine (treated) and the placebo group (untreated) [18], we believed it was still beneficial to investigate the expression profiles of CSF protein biomarkers in subjects of this study to determine if the lamotrigine treatment has any observable effect at protein level. Based upon previous discovery results and an up-to-date literature survey [4,19-21], we selected a total of 26 proteins as potential CSF protein biomarkers for SPMS and developed a mass spectrometric assay for relative quantitation.

## Results

CSF samples were obtained from patient cohorts consisting of 12 SPMS subjects from the National Institute of Neurological Disorders and Stroke (NINDS) at NIH, 10 SPMS subjects from the placebo group involved in clinical trial of lamotrigine, 12 non-inflammatory neurological disorder (NIND) controls from NINDS/NIH and 10 healthy controls from the University of Hawaii (see Table 1). Two biological replicates and three technical replicates were analyzed by SRM mass spectrometry for 26 biomarkers. The protein biomarkers and the peptide sequences monitored, the specific fragmentation transitions, the optimized collision energy (CE) and the start/stop times in a scheduled SRM experiment are listed in Additional file 1: Table S1. Intact horse apomyoglobin was used as an internal standard for quantitation.

**Table 1:** Age, gender and diagnosis group for the 51 subjects

**Group**	**Gender**		**Age (years)**	
	**# Female**	**# Male**	**Mean**	**Range**
HC	2	8	45.4	27-52
NIND	11	1	45.5	29-59
SPMS^(1)^	13	9	53.8	33-69
SPMS (treated) ^(2)^	5	2	53.9	44-60

^(1)^includes 12 NINDS and 10 lamotrigine placebo subjects.

^(2)^treated with lamotrigine.

### Limit of detection, retention time and peak area reproducibility

**Figure 1:**
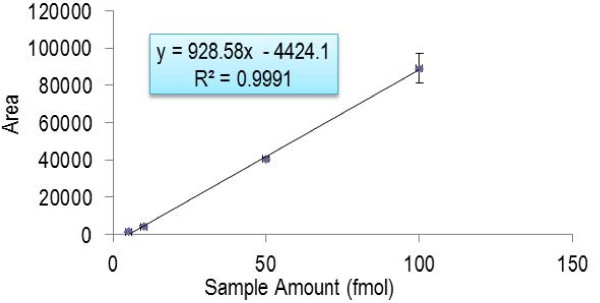
**Standard curve for digested horse apomyoglobin in CSF: x-axis is the concentration of horse apomyoglobin; y-axis is the peak area under the curve detected by LC-MS/MS; insert is the calibration equation with R**^**2**^** as the correlation coefficient**.

**Figure 2:**
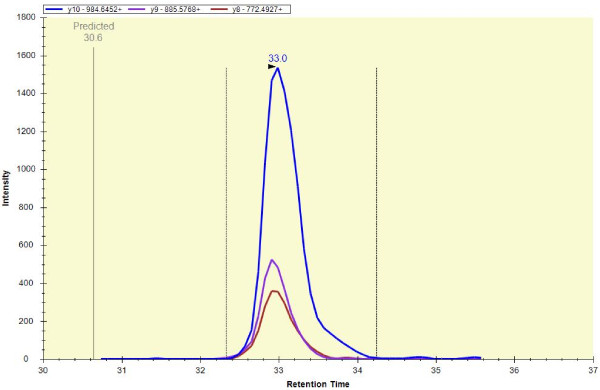
**Representative selected reaction monitoring (SRM) spectrum for the peptide HGTVVLTALGGILK from horse apomyoglobin.** Precursor: 689.9245++, Product Ions: [y10] - 984.6452+, [y9] - 885.5768+ and [y8] - 772.4927+. Each color-coded trace represents one transition. x-axis is time; y-axis is ion intensity.

**Figure 3:**
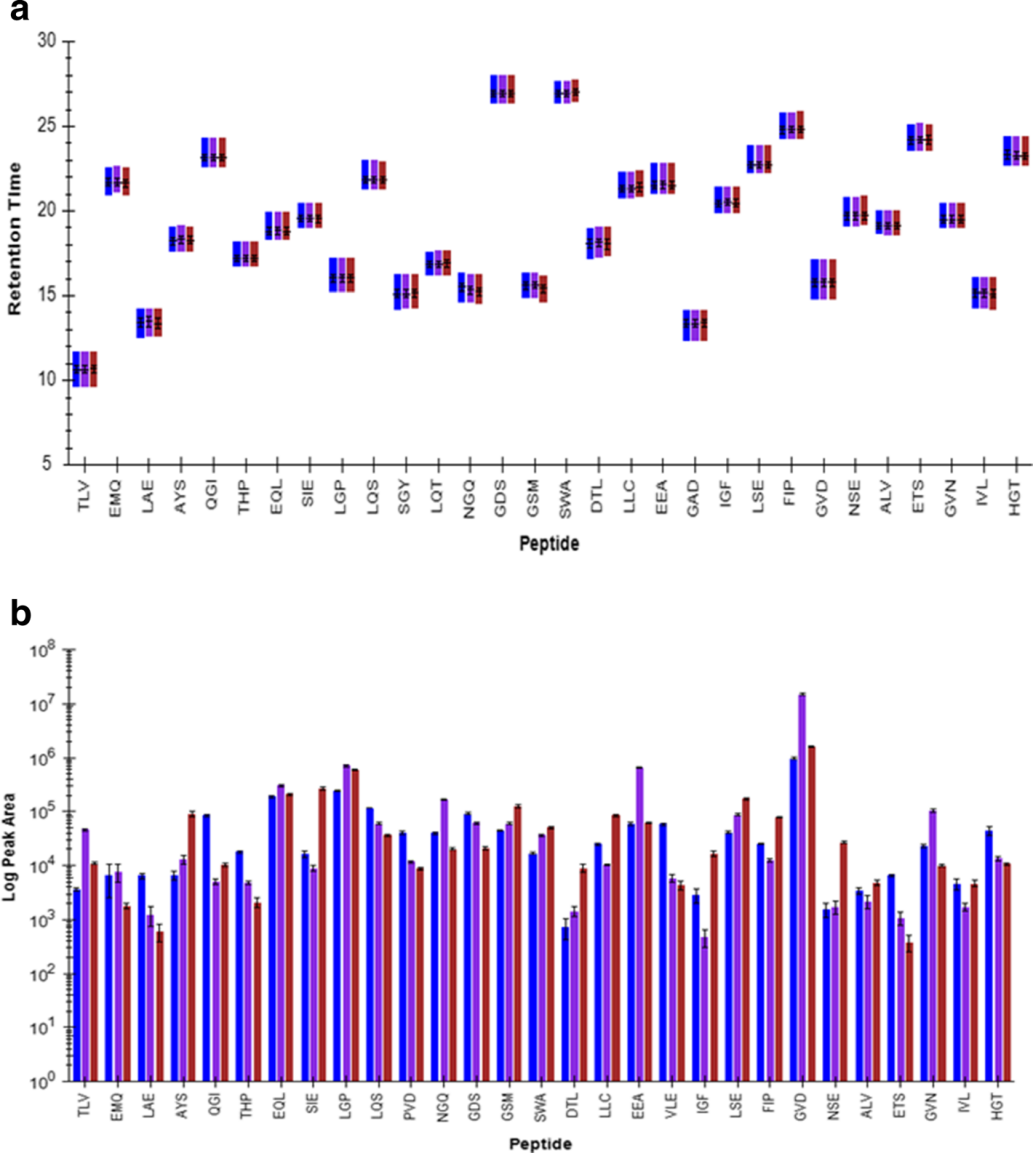
**LC-MS/MS method reproducibility.** (**a**) Retention time reproducibility. Each surrogate peptide is represented on the x-axis by one letter abbreviations of its first three amino acid residues. The three transitions for each peptide are represented in different colors. y-axis is retention time. Data shown are an average of a triplicate. (**b**) Peak area reproducibility. x-axis is peptide; y-axis is peak area in log scale.

The first experiments determined the limit of detection and measurement reproducibility using horse apomyoglobin as internal standard. After optimization of the sample preparation, injection volume, flow rate andchromatography, the final method used 5-fold concentrated CSF, 1μL partial loop injection, 20 μL/min flow rate, a total of 32 min gradient run with a four minutes scheduling window. Intact horse apomyoglobin was serially diluted and added to CSF to obtain the standard curve shown in Figure 1. From this, the LOD was determined as one fmol/μL. These measurements monitored the peptide HGTVVLTALGGILK MH_2_^+2^ ion transitions to the y8, y9 and y10 fragment ions at a retention time of 33 min as shown in Figure 2. Retention time reproducibility (Figure 3a) and peak area reproducibility (Figure 3b) for all surrogate peptides were obtained for their three technical replicates.

### Differential expression profile of selected protein biomarkers in SPMS, NIND and healthy controls

The peak area results for all 26 surrogate peptides, averaged for both biological and technical replicates for all patient and control samples are shown in the Additional file 2: Table S2 along with their standard deviations. Analysis of variance (ANOVA) indicated that five protein biomarkers: amyloid beta A4 protein (A4), alipoprotein E (APOE), kallikrein 6 (KLK6), putative myosin-XVB (MY15B) and pigment epithelium-derived factor (PEDF) were associated with diagnosis (Table 2). Post-hoc multiple comparisons showed that the expression of biomarker MY15B was significantly lower in SPMS than HC. The expression of three biomarkers, A4, APOE and KLL6, were significantly lower in SPMS than NIND group. The expression of biomarker MY15B and PEDF were significantly lower and higher in NIND than HC, respectively.

**Table 2:** **Protein biomarkers and selected principal components with significant difference between three groups (*p*<0.01)**

		**A4**	**APOE**	**KLK6**	**MY15B**	**PEDF**	**RPC2**	**RPC4**
SPMS vs. HC	Ratio^(1)^	0.88	1.15	1.04	**0.33**	1.26		
	p^(2)^	0.7811	0.9358	0.9909	**<0.0001**	0.2423	0.9742	**<0.0001**
SPMS vs. NIND	Ratio^(1)^	**0.55**	**0.58**	**0.62**	0.74	0.81		
	p^(2)^	**0.0084**	**0.008**	**0.004**	0.0502	0.1288	**0.0093**	0.1117
NIND vs. HC	Ratio	1.61	1.97	1.67	**0.44**	**1.55**		
	p^(2)^	0.1178	0.0136	0.0131	**0.0004**	**0.0087**	0.0529	0.0424
F-test (df=2)	p^(3)^	0.0078	0.0032	0.0019	<0.0001	0.0083	0.007	<0.0001

^(1)^ratios of two group medians.

^(2)^*p*-value of pairwise comparison adjusted with Scheffe’s method.

^(3)^*p*-value of global F-test for main effect of diagnosis (10 HC, 12 NIND and 22SPMS).

### Differential expression profile of selected protein biomarkers in lamotrigine trial

Lamotrigine treatment effect in expression was assessed by comparing treated (7 SPMS patients) with untreated (22 SPMS patients) group (Table 3). Eleven proteins were found to have a significant difference in expression between the treated and untreated SPMS group, and all 11 proteins in the treated group had lower expression compared to untreated group, indicating lamotrigine therapy had the effect of decreasing expression of these proteins.

**Table 3:** **Protein biomarkers with significant difference (*p*<0.01) between lamotrigine treated and untreated SPMS group**

**Protein**	***p*-value**^**(1)**^	**Ratio**^**(2)**^
1433F	0.0005	3.37
1433G	0.0017	2.71
AACT	0.0037	2.55
CAD13	0.0018	2.75
TAU	0.0003	2.84
NFH	0.0007	2.25
NFL	0.0011	2.60
NFM	0.0004	4.47
NRCAM	0.0005	2.85
OSTP	0.0062	5.10
SAMP	0.0004	2.64

(1) *p*-value of ANOVA (7 treated vs. 22 untreated SPMS subjects).

(2) The ratios of untreated to treated group median.

### Principal component analysis

Based on the eigenvalue greater than one rule, four principal components accounted for 85.9% of total variance were extracted (RPC1, the first rotated principle component, accounted for 45.9%, RPC2 for 18.6%, RPC3 for 13.7%, and RPC4 for 7.7%), thus, the information in the 26 biomarker variables could be represented by only four components and the four components were used to assess the difference among the three diagnostic categories. According to the component loadings (the correlation coefficients between a component and a protein), most of proteins were highly correlated to only one component. Therefore, the 26 proteins could be clustered into four groups as shown in Table 4. By ANOVA, significant difference between SPMS and NIND was observed in RPC2 and that between SPMS and HC in RPC4 (Table 2). The five biomarkers with significant effect of diagnosis in Table 2 were highly correlated with either RPC2 or RPC4.

**Table 4:** Protein biomarker variable loadings on the four rotated principal components

**Protein**	**RPC1**	**RPC2**	**RPC3**	**RPC4**
1433F	**0.938**	0.123	0.233	0.096
NFL	**0.889**	0.215	0.300	0.136
A2MG	**0.765**	0.502	-0.167	0.114
1433B	**0.742**	0.349	0.088	-0.031
RTN4	**0.560**	-0.366	0.212	0.443
CNTN1	**-0.591**	-0.532	0.513	-0.103
AMD	**-0.804**	0.043	0.213	0.196
1433G	**-0.808**	0.106	-0.335	0.107
CAD13	**-0.819**	-0.246	-0.318	-0.210
AACT	**-0.880**	-0.177	-0.107	-0.114
NRCAM	**-0.885**	-0.239	-0.303	-0.028
KLK6	0.184	**0.941**	0.081	-0.020
VGF	-0.134	**0.935**	-0.008	-0.006
MOG	0.334	**0.878**	0.176	-0.017
PEDF	0.329	**0.780**	0.307	0.087
A4	0.099	**0.740**	0.341	-0.187
APOE	0.655	**0.690**	-0.121	-0.065
SODC	0.227	**0.663**	-0.606	0.249
OSTP	-0.027	-0.037	**0.925**	0.081
SAMP	0.421	0.287	**0.791**	0.257
TAU	0.487	0.243	**0.770**	0.246
NFH	0.552	0.110	**0.764**	0.222
NFM	0.369	0.348	**0.701**	0.185
KLKB1	-0.010	0.117	0.448	**0.864**
AGRIN	0.023	0.112	0.450	**0.862**
MY15B	-0.046	0.298	0.290	**-0.652**

## Discussion

By taking the advantage of high specificity and sensitivity, we have developed a multiplex SRM assay for detecting even low abundant proteins in CSF with relatively simple sample preparation. The platform we have developed here can be easily adapted to other protein biomarker studies. The sensitivity and throughput might be further enhanced using newly developed technologies, such as the dual stage electrodynamic ion funnel interface described by Hossain et al. [22]. Although our assay successfully quantified both high abundant and low abundant proteins in CSF, several proteins which have been previously reported present in CSF were not detected during our initial method development using pooled CSF, such as nidogen-2 (NID2) and nitric oxide synthase (NOS2). Low abundance might be one of the contributing factors, though the intrinsic biological properties of the pooled CSF and the instability of peptides/proteins are also possible. Improvements in sample preparation and optimization of instrument parameters could increase the appearance of low abundant proteins in future studies.

The human proteome is more complex, compared to the genome, considering that each protein can be present in different isoforms even at the same time. However, mass spectrometry-based targeted proteomics has the advantage of specifically quantifying individual protein isoform, which could potentially help us better understand the pathogenesis underlying various diseases. Although we did not include such studies here, the intrinsic specificity of SRM provides the merit to do so if necessary in the future.

Despite CSF’s unique value for neurodegenerative diseases, there were some concerns in the process of searching for protein biomarkers in CSF, such as low protein abundance, difficulties in collecting samples, spontaneous variation through the day, etc. It is essential that CSF collections follow standard operating procedures, including but not limited to the following: all lumbar punctures were carried out at approximately the same time of day, and sample processing (e.g. time to centrifugation, centrifugation speed and time) and storage was handled in a standard way. Our study was carried out among a limited number of patients since the purpose of our study was to validate the feasibility of using SRM for protein biomarker verification. In the future, validation studies on large cohorts of samples originating from healthy controls, relapse-remitting MS (RRMS), SPMS, primary-progressive MS (PPMS), NIND and other inflammatory neurological disease (OIND) controls will determine clinical value of this pilot combinatorial biomarker.

## Conclusions

Using SRM to target a specific set of surrogate biomarker peptides, we established a highly sensitive and specific multiplex assay to simultaneously detect twenty-eight potential protein biomarkers which might be involved in the pathology of SPMS. We quantified the relative levels of each targeted protein using an internal standard horse apomyoglobin and compared their expression profiles among three different cohorts: SPMS, NINDS and healthy controls. ANOVA indicated one protein biomarker- showing significantly different expression between SPMS and HC, and three protein biomarkers showing significantly different expression between SPMS and NIND. It is highly possible that these four proteins play a role in the pathophysiology of SPMS and could potentially benefit the diagnosis, prognosis and/or the development of newer generation treatments. In addition, two proteins were expressed differently between NIND and healthy controls, which could potentially benefit NIND studies. In the lamotrigine trial, although the trial itself came negative with respect to the efficacy of the drug therapy, our analysis suggested that eleven proteins in the treated group had significantly lower expression compared to the untreated group. Since lamotrigine therapy had an effect of decreasing expression for these proteins, it is plausible that these proteins are correlated with other proteins. In fact, our principal component analysis (PCA) revealed that these 26 proteins were correlated, and could be represented by four principal components.Overall, we have established a straightforward mass spectrometry-based platform for CSF protein biomarker development and verification. This platform can be easily adapted to study a broad range of proteins, especially feasible for those of interest to neurodegenerative diseases.

## Methods

### Subjects and clinical samples

CSF samples involved in our study included a coded cohort of 12 untreated SPMS patients and untreated 12 non-inflammatory neurological disorders (NIND) controls from the Neuroimmunology Branch of the National Institutes of Neurological Disorders and Stroke (NIB/NINDS/NIH) collected under natural history protocol 09-N-0032, healthy controls from University of Hawaii, and SPMS patients from a UK double blinded lamotrigine trial (active arm and placebo). All patients signed informed consent and all research procedures were approved by the institutional review boards (IRB) of afore-mentioned institutions.

### Cerebrospinal fluid sample preparation

NIB samples were transported to the laboratory on ice and spun (3000 g x 10 min) within 30 min of collection. Cell-free supernatants were sequentially coded and immediately cryopreserved at −80°C in 500 μl aliquots.

Prior to all sample preparation, we prepared a stock of 1pmol/μL of horse apomyoglobin in deionized water to be used as an internal standard spiked into all CSF samples. Prior to tryptic digestion of the CSF samples used both in method development and then in our cohort screening experiments, 100 μL of 0.1% Rapigest (Waters, Milford, MA) resuspended in 100 mM ammonium bicarbonate, 2 μL 500 mM dithiothreitol (DTT), and 10 μL of the 1pmol/μL stock of the internal standard, horse apomyoglobin, were added to each individual 100 μL CSF aliquot. To reduce protein disulfide bonding, each sample aliquot was heated at 60°C for 30 min. After allowing each sample cool down to room temperature, 10 μL of 200 mM iodoacetamide (IA) was added. Each vial was placed in the dark for 30 min to allow for alkylation of all free protein cysteine residues. 5 μL of trypsin (0.2 μg/μL in 1 mM HCl) was then added to each sample following the DTT reduction and IA alkylation steps. The sample mixtures were incubated at 37°C for 18 h to allow for complete enzymatic digestion of the CSF and the protein standard horse apomyoglobin. Following trypsinization, 2 μL of trifluoroacetic acid was added to the digestion mixture to quench the reaction. The samples were then incubated at 37°C for an additional 45 min. Following this second incubation step, samples were speed-vacuumed to dryness, reconstituted in 0.1% formic acid in deionized water, and desalted using Waters Oasis HLB solid phase extraction cartridges (Waters, Milford, MA) according to the manufacturer’s protocol with a vacuum manifold. To prepare for LC-MS analysis, the eluents from the solid phase extraction were speed-vacuumed to dryness and reconstituted with 20 μL 0.1% formic acid in water. Pooled CSF samples obtained from patients without identified medical records were used during the initial stages of method development. All reagents were added to practice CSF in proportion as described. Diseased CSF and healthy control CSF samples selected for inclusion for our cohort screening experiments were obtained from patients with complete associated medical records. Individual CSF patient samples used during cohort screening were blinded prior to any sample processing. Additionally, sample aliquots were prepared in parallel and as duplicates to minimize experimental error during sample preparation. All reagents were purchased from Sigma if not mentioned specifically.

### HPLC separations

Prior to our selected reaction monitoring assay development efforts, the RP-HPLC gradient profile used for separation of digested CSF samples was first optimized. An Agilent ZORBAX SB-C18 column (150 X 0.5 mm, 5 μm) (Agilent Technologies Inc., Santa Clara, CA, USA) was attached to a Waters Nano-Acquity ultra-high pressure HPLC (Waters, Milford, MA, USA) and placed in the front end of our Thermo Vantage Triple Quadrupole mass spectrometer (Thermo Fisher Scientific, Waltham, MA). Total Ion Chromatograms of the various c18 RP-HPLC separations were acquired in full scan mode acquisition with a scan time of 0.5 s. Ultimately, we selected a 32 min RP-HPLC linear gradient to be used for all subsequent CSF sample separation whereby the injected peptides were eluted with the following gradient: the first 2.4 min post-injection were diverted to waste with a 3% B hold, a linear 3-45% B gradient was performed in the next 22.6 min, a linear 45-90% B gradient ramp was performed in 1 min followed by a 90-100% B linear ramp in 1 min, 100% B was held for 1 min before a linear gradient of 100-3% B was performed in 2 min followed finally by a 3% B hold for 2 min. Solvent A consisted of 0.1% formic acid in water and solvent B consisted of 90% acetonitrile, 10% water and 0.1% formic acid. The flow rate of the Nano-Acquity HPLC was set at 20 μL/min.

### LC-MS/MS method

Intact horse apomyoglobin was chosen as a model protein to establish the LOD of our multiplex SRM LC-MS/MS method. Digested horse apomyoglobin was diluted in serial in digested CSF with concentrations ranging from 1 fmol/μL to 100 fmol/μL. Using a CSF sample with 100 fmol/μL of digested horse apomyoglobin, the following was experimentally determined: the signature tryptic peptide NDIAAK was selected as the surrogate peptide for the apomyoglobin protein, the three most abundant detected transitions of peptide NDIAAK, ions y3, y4, and y5 were selected for detection in Q3, and of those three, the most abundant, y3, was selected for generating a standard curve for this peptide. A serial of apomyoglobin dilutions were then subject to LC-MS/MS analysis with Q1 mass filter targeting detection of the parent ion of NDIAAK and Q3 mass filter targeting detection of the three selected product ions. Ions were introduced into the mass spectrometer via an H-ESI II probe outfitted with a 32 gauge needle (Thermo Fisher Scientific, Waltham, MA, USA). Instrument operating parameters used during acquisition were as follows: capillary temperature of 270°C, vaporizing heat was shut off, sheath gas pressure was set at 10, no auxiliary gas was used, and the spray voltage was set to 4,000 V. Instrument settings in Xcaliber were as follows: positive scan mode, scan widths of 0.004 m/z, scan times of 0.015 s, chrom filters enabled and set at 50, collision gas set at 1.5mTorr, Q1 peak width (FWHM) of 0.7, Q3 peak width of (FWHM) 0.7, and cycle times of 5 s (Xcaliber v 2.1, Thermo Fisher Scientific, Waltham, MA, USA). Following data acquisition, peak areas under the curve were calculated using an open source proteomic software platform Skyline [23] (Skyline v. 1.1, MacCoss Lab, Seattle, WA) and a linear standard curve (y = 5884.7x-9111.7, R^2^ = 0.9998) was generated using Microsoft Excel (Figure 1).

### LC-MS/MS method development and optimization

Using a Waters ultra-high pressure reverse phase high performance liquid chromatography (Waters nanoACQUITY UPLC, Milford, MA, USA) outfitted with an Agilent ZORBAX SB-C18 column (150 X 0.5 mm, 5 μm) (Agilent Technologies Inc., Santa Clara, CA, USA) and coupled to a Thermo Fisher triple quadrupole mass spectrometer (Thermo TSQ Vantage, Thermo Fisher Scientific, Waltham, MA, USA), we designed a multiplexed peptide based selected reaction monitoring (SRM) assay using the RP-HPLC and mass spectrometric settings mentioned in the two previous method sections. Our SRM assay allows for the simultaneous relative quantification of 26 tryptic peptides originating from proteins implicated in SPMS pathology. FASTA files for selected protein biomarkers were downloaded from Uniprot (http://www.uniprot.org) individually and imported into Skyline. The uniqueness of the selected surrogate peptides was confirmed by running individual BLAST search from NCBI website (http://blast.ncbi.nlm.nih.gov/Blast.cgi). Instrumental methods were exported from Skyline into a Thermo Xcaliber 2.1 “EZ SRM” method template with the same settings as used during data acquisition for our LOD determination experiments (Xcaliber v. 2.1, Thermo Fisher Scientific, Milford, MA, USA). To design our SRM assay, we adopted an iterative method development strategy, eliminating poor performing transitions from the SRM assay in a targeted method refinement cycle [23]. Raw data was inspected in the Skyline software platform and the peak areas of each detected transition were normalized to that of horse apomyoglobin peptide HGTVVLTALGGILK. Starting with an initial 1885 transitions for 403 surrogate peptides, we completed seven rounds of iterative method refinement to generate the final SRM assay. The final SRM assay was a scheduled method consisting of 78 transitions for 26 peptides with a scheduling window of four minutes - no more than 50 concurrent transitions were detected at any given time. Reproducibility was confirmed by running four replicates. Both retention time and peak area are highly reproducible (Figure 3a and 3b).

### Data analysis

Box-Cox transformation was applied to CSF values of the 26 biomarkers. Age and gender were considered as covariates, *p*-value of 0.1 was used for covariate selection. Analysis of variance (ANOVA) with or without covariates was performed to assess the differences in the CFS biomarkers values between three cohorts (22 SPMS, 12 NIND and 10 HC), followed by Scheffe’s pair-wise comparison of means. ANOVA was also applied to assess the effect of lamotrigine therapy on the expression of 26 protein biomarkers by comparing 22 SPMS patients with 7 lamotrigine treated SPMS patients.

The relationship between the 26 biomarkers was estimated by Pearson correlation coefficients. Since these biomarkers were found to be closely correlated, principal component analysis (PCA) with Varimax rotation was performed to represent the 26 biomarkers as a set of new orthogonal variables, and subsequently, ANOVA with or without covariates was applied to the selected principal components.

A significant level of 0.01 was used in order to adjust for multiple testing. The statistical analyses were performed using SAS version 9.2.

## Authors’ contributions

YJ and CJ were responsible for setting up and optimizing the selected reaction monitoring (SRM) protocols, carrying the SRM analyses on the triple quadrupole mass spectrometer and obtaining the quantitative results. DC carried out initial work using mass spectrometry in discovery mode to identify potential biomarker targets. PAC helped with design of the clinical aspect of the study, conduct of obtaining CSF from SPMS patients, critical review of the manuscript, and in obtaining partial funding (NMSS TR grant to PAC). TW was responsible for the data and statistical analyses. BB served as PI on NINDS 09-N-0032 protocol under which samples from NINDS SPMS and NIND CSF samples were collected and participated in data analysis, interpretation and critically reviewed the manuscript. SN enrolled patients with SPMS, performed clinical evaluations and conducted all the spinal taps. SG and GG provided the patient samples and data from the Lamotrigine Study. AN conceived of the project, obtained funding, coordinated various sites, supervised sample processing and analysis, assisted with data analysis and interpretation and reviewed the manuscript. RJC is Director of the Middle Atlantic Mass Spectrometry Laboratory and had overall responsibility for the conduct and interpretation of the mass spectral measurements. All authors read and approved the final manuscript.

## Supplementary Material

Additional file 1 **Table S1.** Peptides and transitions used in SRM (DOC 115 kb) Click here for file

Additional file 2 **Table S2.** Peak areas averaged for both biological and technical replicates and standard deviations for the 26 surrogate peptides. (DOC 568 kb) Click here for file
