# Supplementary material for: Development of protein biomarkers in cerebrospinal fluid for secondary progressive multiple sclerosis using selected reaction monitoring mass spectrometry (SRM-MS)
Source: Clin Proteomics. 2012 Jul 30;9(1):9. doi: 10.1186/1559-0275-9-9 (PMC3466133; doi:10.1186/1559-0275-9-9)
Supplement: Additional file 1 — Table S1. Peptides and transitions used in SRM (DOC 115 kb) [file 1559-0275-9-9-S1.doc]

| Supplement Table 1. Peptides and transitions used in SRM | | | | | | | |
| --- | --- | --- | --- | --- | --- | --- | --- |
| Protein | Peptide | Parent Ion m/z | Product Ion m/z | Transi-tion | CE | Start Time/min | End Time/min |
| Superoxide dismutase [Cu-Zn] | TLVVHEK | 413.2451 | 724.4352 | y6 | 15.2 | 9.37 | 13.37 |
| sp|P00441|SODC_HUMAN |  |  | 611.3511 | y5 | 15.2 | 9.37 | 13.37 |
|  |  |  | 512.2827 | y4 | 15.2 | 9.37 | 13.37 |
| Alpha-2-macroglobulin | QGIPFFGQVR | 574.8142 | 850.4570 | y7 | 20 | 22.12 | 26.12 |
| sp|P01023|A2MG_HUMAN |  |  | 753.4042 | y6 | 20 | 22.12 | 26.12 |
|  |  |  | 606.3358 | y5 | 20 | 22.12 | 26.12 |
| Amyloid beta A4 protein | THPHFVIPYR | 633.8407 | 1028.5676 | y8 | 21.8 | 16.11 | 20.11 |
| sp|P05067-2|A4_HUMAN |  |  | 794.4559 | y6 | 21.8 | 16.11 | 20.11 |
|  |  |  | 647.3875 | y5 | 21.8 | 16.11 | 20.11 |
| Alpha-1-antichymotrypsin | EQLSLLDR | 487.2693 | 716.4301 | y6 | 17.4 | 17.8 | 21.8 |
| sp|P01011|AACT_HUMAN |  |  | 603.3461 | y5 | 17.4 | 17.8 | 21.8 |
|  |  |  | 403.2300 | y3 | 17.4 | 17.8 | 21.8 |
| Agrin | SIESTLDDLFR | 648.3275 | 1095.5317 | y9 | 22.2 | 18.51 | 22.51 |
| sp|O00468|AGRIN_HUMAN |  |  | 778.4094 | y6 | 22.2 | 18.51 | 22.51 |
|  |  |  | 665.3253 | y5 | 22.2 | 18.51 | 22.51 |
| Apolipoprotein E | LGPLVEQGR | 484.7798 | 701.3941 | y6 | 17.3 | 15.08 | 19.08 |
| sp|P02649|APOE_HUMAN |  |  | 588.3100 | y5 | 17.3 | 15.08 | 19.08 |
|  |  |  | 489.2416 | y4 | 17.3 | 15.08 | 19.08 |
| Pigment epithelium-derived factor | LQSLFDSPDFSK | 692.3432 | 1142.5364 | y10 | 23.6 | 20.86 | 24.86 |
| sp|P36955|PEDF_HUMAN |  |  | 942.4203 | y8 | 23.6 | 20.86 | 24.86 |
|  |  |  | 795.3519 | y7 | 23.6 | 20.86 | 24.86 |
| Microtubule-associated protein tau | PVDLSK | 329.6921 | 347.2289 | y3 | 12.7 | 14.75 | 18.75 |
| sp|P10636|TAU_HUMAN |  |  | 234.1448 | y2 | 12.7 | 14.75 | 18.75 |
|  |  |  | 147.1128 | y1 | 12.7 | 14.75 | 18.75 |
| Peptidyl-glycine alpha-amidating monooxygenase isoform 2  sp|P19021-2|AMD_HUMAN | NGQWTLIGR | 522.7829 | 458.3085 | y4 | 18.5 | 13.77 | 17.77 |
|  |  |  | 345.2245 | y3 | 18.5 | 13.77 | 17.77 |
|  |  |  | 232.1404 | y2 | 18.5 | 13.77 | 17.77 |
| Osteopontin | GDSVVYGLR | 483.2562 | 607.3562 | y5 | 17.3 | 25.6 | 29.6 |
| sp|P10451|OSTP_HUMAN |  |  | 508.2878 | y4 | 17.3 | 25.6 | 29.6 |
|  |  |  | 345.2245 | y3 | 17.3 | 25.6 | 29.6 |
| Neuronal cell adhesion molecule | GSMVSFECK | 522.7281 | 769.3549 | y6 | 18.5 | 13.84 | 17.84 |
| sp|Q92823|NRCAM_HUMAN |  |  | 670.2865 | y5 | 18.5 | 13.84 | 17.84 |
|  |  |  | 583.2545 | y4 | 18.5 | 13.84 | 17.84 |
| Reticulon-4 | DTLLPDEVSTLSK | 709.3747 | 1088.5834 | y10 | 24.1 | 16.57 | 20.57 |
| sp|Q9NQC3|RTN4_HUMAN |  |  | 975.4993 | y9 | 24.1 | 16.57 | 20.57 |
|  |  |  | 535.3086 | y5 | 24.1 | 16.57 | 20.57 |
| Neurofilament medium polypeptide | EEAVAEVVTITK | 644.8534 | 789.4716 | y7 | 22.1 | 20.59 | 24.59 |
| sp|P07197|NFM_HUMAN |  |  | 660.4291 | y6 | 22.1 | 20.59 | 24.59 |
| NFM |  |  | 561.3606 | y5 | 22.1 | 20.59 | 24.59 |
| Neurofilament light polypeptide  sp|P07196|NFL_HUMAN | VLEAELLVLR | 577.8608 | 942.5619 | y8 | 20.1 | 17.41 | 21.41 |
|  |  |  | 500.3555 | y4 | 20.1 | 17.41 | 21.41 |
|  |  |  | 387.2714 | y3 | 20.1 | 17.41 | 21.41 |
| Neurofilament heavy polypeptide | IGFGPIPFSLPEGLPK | 834.9716 | 1294.7406 | y12 | 27.8 | 19.32 | 23.32 |
| sp|P12036|NFH_HUMAN |  |  | 1084.6037 | y10 | 27.8 | 19.32 | 23.32 |
|  |  |  | 753.4505 | y7 | 27.8 | 19.32 | 23.32 |
| 14-3-3 protein beta/alpha | EMQPTHPIR | 554.7820 | 720.4151 | y6 | 19.4 | 20.39 | 24.39 |
| sp|P31946|1433B_HUMAN |  |  | 623.3624 | y5 | 19.4 | 20.39 | 24.39 |
|  |  |  | 522.3147 | y4 | 19.4 | 20.39 | 24.39 |
| 14-3-3 protein eta | LAEQAER | 408.7141 | 503.2572 | y4 | 15 | 12.2 | 16.2 |
| sp|Q04917|1433F_HUMAN |  |  | 375.1987 | y3 | 15 | 12.2 | 16.2 |
|  |  |  | 304.1615 | y2 | 15 | 12.2 | 16.2 |
| 14-3-3 protein gamma | AYSEAHEISK | 567.7749 | 813.4101 | y7 | 19.8 | 15.24 | 19.24 |
| sp|P61981|1433G_HUMAN |  |  | 684.3675 | y6 | 19.8 | 15.24 | 19.24 |
|  |  |  | 613.3304 | y5 | 19.8 | 15.24 | 19.24 |
| Kallikrein-6 | LSELIQPLPLER | 704.4139 | 965.5778 | y8 | 23.9 | 21.73 | 25.73 |
| sp|Q92876|KLK6_HUMAN |  |  | 852.4938 | y7 | 23.9 | 21.73 | 25.73 |
|  |  |  | 724.4352 | y6 | 23.9 | 21.73 | 25.73 |
| Contactin-1 | FIPLIPIPER | 597.8659 | 934.5720 | y8 | 20.7 | 23.91 | 27.91 |
| sp|Q12860|CNTN1_HUMAN |  |  | 724.4352 | y6 | 20.7 | 23.91 | 27.91 |
|  |  |  | 611.3511 | y5 | 20.7 | 23.91 | 27.91 |
| Cadherin-13 | GVDQEPK | 386.6954 | 501.2667 | y4 | 14.4 | 14.76 | 18.76 |
| sp|P55290|CAD13_HUMAN |  |  | 244.1656 | y2 | 14.4 | 14.76 | 18.76 |
|  |  |  | 147.1128 | y1 | 14.4 | 14.76 | 18.76 |
| Neurosecretory protein VGF | NSEPQDEGELFQGVDPR | 958.9347 | 818.4155 | y7 | 31.6 | 18.73 | 22.73 |
| sp|O15240|VGF_HUMAN |  |  | 671.3471 | y6 | 31.6 | 18.73 | 22.73 |
|  |  |  | 272.1717 | y2 | 31.6 | 18.73 | 22.73 |
| Myelin-oligodendrocyte glycoprotein | ALVGDEVELPCR | 679.3427 | 1074.4884 | y9 | 23.2 | 17.89 | 21.89 |
| sp|Q16653|MOG_HUMAN |  |  | 545.2864 | y4 | 23.2 | 17.89 | 21.89 |
|  |  |  | 432.2024 | y3 | 23.2 | 17.89 | 21.89 |
| Putative myosin-XVB | ETSEEAEDRPYQPK | 839.8814 | 903.4683 | y7 | 28 | 27.01 | 31.01 |
| sp|Q96JP2|MY15B_HUMAN |  |  | 535.2875 | y4 | 28 | 27.01 | 31.01 |
|  |  |  | 372.2241 | y3 | 28 | 27.01 | 31.01 |
| Kallikrein | GVNVCQETCTK | 648.2896 | 1025.4390 | y8 | 22.2 | 18.53 | 22.53 |
| sp|P03952|KLKB1_HUMAN |  |  | 926.3706 | y7 | 22.2 | 18.53 | 22.53 |
|  |  |  | 509.2388 | y4 | 22.2 | 18.53 | 22.53 |
| Serum amyloid P-component | IVLGQEQDSYGGK | 697.3515 | 883.3792 | y8 | 23.7 | 13.91 | 17.91 |
| sp|P02743|SAMP_HUMAN |  |  | 754.3366 | y7 | 23.7 | 13.91 | 17.91 |
|  |  |  | 511.2511 | y5 | 23.7 | 13.91 | 17.91 |
