# Supplementary material for: Development of protein biomarkers in cerebrospinal fluid for secondary progressive multiple sclerosis using selected reaction monitoring mass spectrometry (SRM-MS)
Source: Clin Proteomics. 2012 Jul 30;9(1):9. doi: 10.1186/1559-0275-9-9 (PMC3466133; doi:10.1186/1559-0275-9-9)
Supplement: Additional file 2 — Table S2. Peak areas averaged for both biological and technical replicates and standard deviations for the 26 surrogate peptides. (DOC 568 kb) [file 1559-0275-9-9-S2.doc]

| Supplement Table 2. Peak areas averaged for both biological and technical replicates and standard deviations for the 26 surrogate peptides. | | | | | | | | | | | | | | |
| --- | --- | --- | --- | --- | --- | --- | --- | --- | --- | --- | --- | --- | --- | --- |
| DG | Treat-ment | Sample Code | MOG | Std | 1433G | Std | RTN4 | Std | NFM | Std | 1433B | Std | AACT | Std |
| SPMS | N | A4 | 22.84 | 2.04 | 2.93 | 1.27 | 25.09 | 4.53 | 2177.26 | 183.32 | 57.74 | 2.88 | 222.63 | 20.67 |
| SPMS | N | A79 | 28.42 | 2.12 | 1.58 | 0.99 | 20.25 | 3.39 | 911.54 | 10.73 | 45.54 | 4.53 | 56.79 | 2.96 |
| SPMS | N | A37 | 18.80 | 2.41 | 3.27 | 1.01 | 25.94 | 12.20 | 2048.50 | 426.66 | 45.17 | 3.17 | 104.27 | 6.86 |
| SPMS | N | A78 | 14.64 | 2.86 | 3.15 | 1.91 | 24.94 | 13.77 | 2181.42 | 65.37 | 35.52 | 7.01 | 103.58 | 7.67 |
| SPMS | N | A30 | 22.85 | 3.28 | 3.95 | 1.68 | 39.21 | 4.87 | 4527.62 | 267.59 | 45.00 | 3.65 | 65.97 | 7.55 |
| SPMS | N | A26 | 15.51 | 3.06 | 7.32 | 3.08 | 47.33 | 6.52 | 2768.50 | 2096.24 | 37.17 | 8.51 | 171.66 | 14.25 |
| SPMS | N | A1_59 | 23.87 | 2.16 | 2.25 | 1.19 | 15.15 | 6.76 | 973.66 | 453.36 | 35.71 | 10.92 | 87.80 | 2.04 |
| SPMS | N | A2_59 | 29.89 | 3.51 | 1.30 | 0.87 | 14.90 | 3.12 | 980.02 | 654.51 | 34.86 | 16.71 | 79.77 | 13.76 |
| SPMS | N | A1_70 | 14.62 | 2.64 | 5.08 | 1.61 | 34.59 | 2.98 | 1077.10 | 182.62 | 22.05 | 5.47 | 132.49 | 3.90 |
| SPMS | N | A2_70 | 10.79 | 2.21 | 6.40 | 0.98 | 39.97 | 3.94 | 799.87 | 556.42 | 27.53 | 8.52 | 108.38 | 13.54 |
| SPMS | N | A1_62 | 15.91 | 1.37 | 3.49 | 1.85 | 31.16 | 5.60 | 1780.25 | 854.21 | 28.06 | 5.23 | 62.22 | 13.58 |
| SPMS | N | A2_62 | 18.95 | 1.84 | 2.68 | 1.94 | 29.58 | 5.20 | 1371.12 | 362.24 | 29.56 | 4.71 | 72.31 | 10.53 |
| SPMS | N | A1_90 | 21.84 | 2.20 | 3.55 | 1.16 | 24.95 | 5.45 | 1134.99 | 41.28 | 38.57 | 2.57 | 68.40 | 5.55 |
| SPMS | N | A2_90 | 23.90 | 4.06 | 5.05 | 3.60 | 25.12 | 10.38 | 866.31 | 97.55 | 32.17 | 8.39 | 76.91 | 4.25 |
| SPMS | N | NIB-156 | 25.15 | 5.17 | 12.95 | 6.11 | 30.25 | 6.16 | 6906.67 | 185.64 | 28.12 | 26.78 | 173.81 | 14.50 |
| SPMS | N | NIB-164 | 59.21 | 8.39 | 7.10 | 4.55 | 29.13 | 16.49 | 2480.90 | 51.08 | 67.24 | 3.49 | 198.48 | 6.23 |
| SPMS | N | NIB-169 | 26.49 | 0.43 | 3.45 | 0.67 | 27.93 | 11.95 | 5436.32 | 175.30 | 23.88 | 17.45 | 187.23 | 7.43 |
| SPMS | N | NIB-176 | 28.82 | 0.59 | 12.49 | 4.26 | 7.26 | 5.68 | 3381.83 | 34.71 | 40.49 | 5.35 | 150.10 | 1.67 |
| SPMS | N | NIB-187 | 37.57 | 2.38 | 667.92 | 24.84 | 67.03 | 11.08 | 2882.46 | 213.02 | 94.08 | 21.60 | 3149.39 | 8.75 |
| SPMS | N | NIB-188 | 46.71 | 0.35 | 3.52 | 1.50 | 17.35 | 3.20 | 3476.37 | 72.66 | 33.32 | 25.61 | 226.13 | 9.32 |
| SPMS | N | NIB-189 | 36.59 | 7.28 | 7.34 | 1.47 | 50.76 | 6.32 | 10135.67 | 408.99 | 74.22 | 11.09 | 281.48 | 25.77 |
| SPMS | N | NIB-214 | 55.79 | 5.02 | 5.44 | 2.14 | 41.28 | 6.09 | 10706.40 | 1333.55 | 82.58 | 23.08 | 224.60 | 29.91 |
| SPMS | N | NIB-077 | 34.42 | 1.96 | 3.91 | 1.34 | 14.36 | 12.40 | 2546.40 | 180.55 | 42.28 | 2.51 | 107.61 | 10.41 |
| SPMS | N | NIB-088 | 60.88 | 5.97 | 782.59 | 9.04 | 44.01 | 25.66 | 10120.89 | 363.20 | 153.53 | 10.63 | 1987.17 | 34.45 |
| SPMS | N | NIB-098 | 61.50 | 4.73 | 2.69 | 1.84 | 18.20 | 8.72 | 4191.96 | 20.89 | 48.38 | 2.59 | 97.34 | 2.77 |
| SPMS | N | NIB-107 | 73.25 | 3.16 | 8.01 | 2.26 | 26.70 | 25.09 | 9478.47 | 136.47 | 61.49 | 46.10 | 222.22 | 1.93 |
| HC | N | 60167 | 9.97 | 4.75 | 2.64 | 1.63 | 19.61 | 1.75 | 644.14 | 213.32 | 24.70 | 4.58 | 37.54 | 4.26 |
| HC | N | 80155 | 27.93 | 4.06 | 2.17 | 1.33 | 16.92 | 6.52 | 1473.90 | 183.99 | 26.09 | 3.98 | 68.93 | 6.88 |
| HC | N | 100263 | 20.08 | 2.53 | 3.96 | 2.45 | 32.08 | 15.36 | 3068.98 | 198.46 | 47.79 | 6.18 | 110.45 | 8.58 |
| HC | N | 100129 | 29.51 | 2.94 | 1.48 | 1.56 | 12.41 | 11.85 | 2956.97 | 1731.89 | 46.87 | 7.37 | 41.22 | 7.06 |
| HC | N | 60023 | 27.44 | 2.70 | 6.60 | 2.87 | 6.36 | 4.53 | 1167.57 | 29.82 | 32.43 | 5.63 | 92.84 | 5.94 |
| HC | N | 60163 | 30.49 | 5.67 | 7.11 | 2.13 | 17.43 | 1.19 | 4514.64 | 109.78 | 50.93 | 7.54 | 137.47 | 4.82 |
| HC | N | 90092 | 50.76 | 3.38 | 1.69 | 1.45 | 9.23 | 4.76 | 2094.28 | 11.03 | 53.99 | 6.79 | 96.78 | 4.77 |
| HC | N | 90149 | 38.04 | 1.27 | 6.16 | 6.12 | 25.88 | 3.80 | 5289.81 | 68.09 | 43.76 | 5.95 | 123.92 | 3.08 |
| HC | N | 70118 | 32.47 | 2.24 | 8.93 | 4.58 | 29.14 | 5.20 | 4044.09 | 126.83 | 35.49 | 1.11 | 134.40 | 8.11 |
| HC | N | 40010 | 41.65 | 5.17 | 2.58 | 1.66 | 32.89 | 14.59 | 5641.00 | 614.93 | 44.43 | 6.77 | 93.19 | 4.16 |
| NIND | N | NIB-091 | 32.94 | 1.70 | 334.21 | 41.80 | 26.03 | 4.46 | 2210.20 | 263.19 | 77.28 | 14.78 | 889.02 | 76.56 |
| NIND | N | NIB-103 | 47.44 | 0.27 | 1.17 | 1.06 | 15.78 | 10.81 | 11098.83 | 146.32 | 59.71 | 11.91 | 97.00 | 4.01 |
| NIND | N | NIB-114 | 53.91 | 7.33 | 502.48 | 17.82 | 43.50 | 19.14 | 4225.05 | 269.40 | 66.82 | 14.51 | 2098.23 | 5.80 |
| NIND | N | NIB-134 | 58.83 | 4.73 | 2.23 | 1.82 | 7.37 | 0.79 | 1266.68 | 21.18 | 54.06 | 2.80 | 132.35 | 10.41 |
| NIND | N | NIB-137 | 66.03 | 7.56 | 422.57 | 25.90 | 19.03 | 17.47 | 1796.98 | 20.40 | 95.11 | 1.69 | 1179.82 | 13.84 |
| NIND | N | NIB-144 | 64.44 | 8.92 | 4.40 | 2.01 | 7.48 | 20.72 | 8584.31 | 143.65 | 66.60 | 0.81 | 144.52 | 5.09 |
| NIND | N | NIB-148 | 60.80 | 3.23 | 432.74 | 19.08 | 37.92 | 4.59 | 2764.56 | 121.39 | 93.16 | 4.61 | 1576.54 | 70.89 |
| NIND | N | NIB-165 | 53.11 | 3.91 | 4.95 | 2.21 | 23.69 | 27.54 | 5141.21 | 88.92 | 21.98 | 16.71 | 98.56 | 5.96 |
| NIND | N | NIB-206 | 27.83 | 2.02 | 1.36 | 1.32 | 14.24 | 10.81 | 4222.61 | 223.93 | 36.15 | 12.77 | 92.97 | 1.81 |
| NIND | N | NIB-208 | 42.43 | 8.15 | 2.50 | 3.05 | 27.89 | 17.41 | 2143.02 | 21.21 | 30.55 | 3.75 | 47.09 | 2.01 |
| NIND | N | NIB-212 | 34.42 | 2.49 | 4.71 | 1.39 | 30.00 | 7.69 | 4717.89 | 58.32 | 46.03 | 4.60 | 120.05 | 6.04 |
| NIND | N | NIB-215 | 46.13 | 5.29 | 3.51 | 1.39 | 45.53 | 26.03 | 4648.874 | 53.9126 | 36.72 | 7.21 | 56.90 | 6.97 |
| SPMS | Y | B52 | 29.06 | 3.92 | 0.26 | 0.50 | 6.63 | 6.20 | 289.71 | 277.56 | 13.78 | 15.57 | 112.05 | 47.04 |
| SPMS | Y | B64 | 18.59 | 2.46 | 1.66 | 0.92 | 21.11 | 2.05 | 801.63 | 414.06 | 28.69 | 6.58 | 63.05 | 7.55 |
| SPMS | Y | B80 | 26.90 | 2.70 | 1.45 | 0.80 | 12.73 | 6.33 | 611.66 | 89.97 | 33.34 | 5.89 | 67.24 | 12.75 |
| SPMS | Y | B71 | 17.85 | 5.03 | 4.82 | 1.44 | 55.93 | 5.02 | 2028.31 | 763.50 | 48.70 | 4.48 | 111.76 | 9.10 |
| SPMS | Y | B1_96 | 25.03 | 2.75 | 0.99 | 0.47 | 9.09 | 5.55 | 307.20 | 57.51 | 28.11 | 7.86 | 47.55 | 5.46 |
| SPMS | Y | B2_96 | 27.22 | 3.38 | 1.46 | 0.77 | 8.91 | 5.74 | 492.42 | 41.52 | 31.34 | 3.19 | 46.38 | 2.64 |
| SPMS | Y | B1_98 | 24.22 | 4.57 | 1.54 | 1.07 | 8.72 | 3.27 | 347.43 | 111.03 | 30.78 | 6.96 | 52.79 | 30.91 |
| SPMS | Y | B2_98 | 21.97 | 2.15 | 2.22 | 2.02 | 14.52 | 11.38 | 632.76 | 194.52 | 25.86 | 4.58 | 55.40 | 3.68 |
| SPMS | Y | B1_117 | 31.29 | 9.97 | 2.35 | 1.12 | 16.85 | 16.81 | 5161.61 | 1633.44 | 38.76 | 26.70 | 58.12 | 44.09 |
| SPMS | Y | B2_117 | 22.29 | 5.06 | 1.80 | 0.58 | 11.03 | 7.10 | 1570.22 | 486.54 | 25.32 | 9.85 | 57.82 | 6.26 |
|  |  |  |  |  |  |  |  |  |  |  |  |  |  |  |
| DG | Treat-ment | Sample Code | MY15B | Std | CNTN1 | Std | NFL | Std | OSTP | Std | NRCAM | Std | CAD13 | Std |
| SPMS | N | A4 | 45.43 | 2.89 | 69.43 | 8.96 | 11.89 | 1.79 | 21.37 | 2.26 | 12.47 | 1.55 | 14025.30 | 1253.24 |
| SPMS | N | A79 | 40.51 | 5.06 | 68.41 | 4.29 | 7.53 | 1.22 | 14.72 | 2.36 | 9.43 | 2.39 | 9349.31 | 553.58 |
| SPMS | N | A37 | 28.77 | 3.01 | 52.79 | 3.11 | 14.96 | 0.85 | 25.79 | 10.64 | 17.24 | 2.28 | 18352.69 | 662.11 |
| SPMS | N | A78 | 58.90 | 8.01 | 31.50 | 5.11 | 14.21 | 0.58 | 33.43 | 7.89 | 15.09 | 1.41 | 16309.11 | 1043.22 |
| SPMS | N | A30 | 42.33 | 2.73 | 53.96 | 8.43 | 18.64 | 1.31 | 37.51 | 7.34 | 18.33 | 2.58 | 22022.16 | 2398.03 |
| SPMS | N | A26 | 23.15 | 1.47 | 54.67 | 10.94 | 23.82 | 1.81 | 67.26 | 6.02 | 27.46 | 3.17 | 29946.18 | 3636.75 |
| SPMS | N | A1_59 | 57.19 | 11.56 | 49.63 | 6.86 | 11.29 | 1.88 | 6.63 | 2.82 | 11.21 | 1.76 | 14223.13 | 546.10 |
| SPMS | N | A2_59 | 37.78 | 10.83 | 67.26 | 19.23 | 9.50 | 1.80 | 6.02 | 4.70 | 11.86 | 2.32 | 12969.91 | 2003.62 |
| SPMS | N | A1_70 | 43.31 | 3.88 | 33.93 | 2.65 | 24.26 | 1.95 | 10.21 | 1.16 | 22.91 | 3.03 | 26966.72 | 2236.83 |
| SPMS | N | A2_70 | 26.92 | 4.94 | 36.06 | 4.36 | 18.63 | 1.36 | 52.21 | 9.14 | 18.35 | 2.96 | 20361.50 | 2667.28 |
| SPMS | N | A1_62 | 27.60 | 5.32 | 37.03 | 5.27 | 12.60 | 1.43 | 24.85 | 8.57 | 17.92 | 2.03 | 16332.02 | 1360.16 |
| SPMS | N | A2_62 | 39.83 | 3.68 | 42.53 | 4.84 | 15.73 | 3.95 | 27.14 | 1.75 | 19.07 | 2.40 | 17821.76 | 3904.15 |
| SPMS | N | A1_90 | 44.00 | 4.88 | 57.35 | 5.11 | 14.19 | 1.20 | 16.24 | 2.77 | 18.10 | 3.11 | 16433.61 | 238.58 |
| SPMS | N | A2_90 | 30.43 | 3.52 | 57.51 | 7.95 | 14.13 | 1.19 | 21.30 | 9.38 | 16.25 | 1.35 | 16562.15 | 1161.48 |
| SPMS | N | NIB-156 | 40.34 | 4.62 | 40.56 | 4.30 | 41.08 | 2.05 | 106.51 | 9.02 | 53.20 | 9.14 | 51291.13 | 2651.46 |
| SPMS | N | NIB-164 | 39.14 | 0.62 | 47.85 | 3.54 | 26.71 | 1.18 | 119.76 | 2.94 | 37.42 | 7.01 | 29078.85 | 2117.90 |
| SPMS | N | NIB-169 | 55.66 | 2.05 | 38.08 | 2.52 | 15.64 | 1.37 | 46.69 | 2.39 | 13.42 | 10.99 | 30336.87 | 176.05 |
| SPMS | N | NIB-176 | 55.21 | 1.79 | 40.01 | 0.95 | 25.28 | 4.50 | 63.96 | 3.82 | 26.17 | 14.00 | 24481.79 | 2392.40 |
| SPMS | N | NIB-187 | 54.04 | 0.94 | 743.41 | 63.15 | 90.89 | 8.73 | 19.86 | 11.64 | 677.66 | 22.12 | 181842.37 | 919.90 |
| SPMS | N | NIB-188 | 50.50 | 3.63 | 93.04 | 5.44 | 42.60 | 2.09 | 122.29 | 7.65 | 40.16 | 4.11 | 46324.85 | 1304.83 |
| SPMS | N | NIB-189 | 39.22 | 1.75 | 50.82 | 5.79 | 37.00 | 4.46 | 118.55 | 8.76 | 41.23 | 16.65 | 51531.45 | 1561.42 |
| SPMS | N | NIB-214 | 51.42 | 8.17 | 60.88 | 8.93 | 28.50 | 4.08 | 65.94 | 8.30 | 36.07 | 3.55 | 37811.04 | 6003.71 |
| SPMS | N | NIB-077 | 40.53 | 6.60 | 62.07 | 2.96 | 20.01 | 8.72 | 70.02 | 7.22 | 26.86 | 1.62 | 30538.93 | 612.37 |
| SPMS | N | NIB-088 | 50.49 | 7.30 | 915.17 | 42.73 | 93.90 | 6.85 | 11.99 | 1.50 | 709.01 | 19.06 | 246538.42 | 2102.36 |
| SPMS | N | NIB-098 | 58.14 | 1.97 | 46.87 | 1.77 | 20.86 | 1.32 | 60.57 | 1.10 | 25.97 | 4.89 | 23873.32 | 779.71 |
| SPMS | N | NIB-107 | 36.18 | 0.47 | 89.34 | 4.85 | 40.21 | 3.25 | 100.09 | 3.65 | 66.73 | 2.84 | 46864.57 | 2014.97 |
| HC | N | 60167 | 31.55 | 4.92 | 42.42 | 1.93 | 6.44 | 1.49 | 19.97 | 3.56 | 9.01 | 0.99 | 8560.68 | 689.62 |
| HC | N | 80155 | 157.46 | 13.73 | 63.40 | 5.08 | 10.81 | 0.41 | 15.96 | 5.49 | 12.21 | 1.77 | 12476.36 | 1364.75 |
| HC | N | 100263 | 112.94 | 6.66 | 56.17 | 3.46 | 22.87 | 2.39 | 82.89 | 4.31 | 23.11 | 3.67 | 26904.20 | 412.84 |
| HC | N | 100129 | 138.60 | 14.01 | 80.15 | 2.30 | 7.72 | 1.33 | 22.55 | 8.13 | 8.22 | 2.04 | 8679.17 | 1559.65 |
| HC | N | 60023 | 106.13 | 2.07 | 33.03 | 2.92 | 16.62 | 2.67 | 81.98 | 5.10 | 33.64 | 4.26 | 18977.98 | 1907.32 |
| HC | N | 60163 | 208.49 | 12.19 | 35.64 | 2.46 | 14.70 | 1.24 | 67.73 | 5.89 | 19.30 | 23.86 | 18819.11 | 972.49 |
| HC | N | 90092 | 200.44 | 5.96 | 79.46 | 5.62 | 16.36 | 2.38 | 50.48 | 15.87 | 21.82 | 2.48 | 23078.42 | 213.93 |
| HC | N | 90149 | 69.29 | 4.60 | 47.36 | 2.26 | 21.25 | 2.99 | 56.01 | 3.07 | 28.27 | 9.22 | 28647.02 | 816.71 |
| HC | N | 70118 | 92.32 | 8.77 | 41.61 | 1.84 | 34.05 | 3.82 | 104.53 | 15.72 | 35.70 | 14.02 | 41057.34 | 500.95 |
| HC | N | 40010 | 133.06 | 10.74 | 49.04 | 11.55 | 19.23 | 2.70 | 102.23 | 135.56 | 24.60 | 8.14 | 24734.42 | 1538.01 |
| NIND | N | NIB-091 | 56.27 | 4.38 | 782.57 | 142.42 | 50.99 | 1.58 | 12.07 | 6.09 | 429.04 | 34.96 | 98127.73 | 12556.94 |
| NIND | N | NIB-103 | 112.70 | 11.48 | 69.16 | 6.34 | 21.95 | 4.70 | 28.06 | 2.47 | 19.01 | 3.31 | 27230.83 | 1788.45 |
| NIND | N | NIB-114 | 70.49 | 11.45 | 783.55 | 51.81 | 94.48 | 3.05 | 12.99 | 0.14 | 615.47 | 0.02 | 158281.90 | 3657.07 |
| NIND | N | NIB-134 | 52.79 | 1.30 | 92.86 | 9.29 | 21.83 | 5.52 | 45.03 | 9.52 | 36.63 | 6.34 | 24768.25 | 149.07 |
| NIND | N | NIB-137 | 43.29 | 1.56 | 1025.36 | 26.10 | 57.64 | 6.08 | 11.37 | 2.36 | 475.00 | 50.27 | 128506.34 | 767.23 |
| NIND | N | NIB-144 | 50.61 | 2.17 | 100.42 | 6.33 | 26.78 | 2.01 | 56.69 | 8.45 | 39.39 | 7.50 | 38228.06 | 1078.38 |
| NIND | N | NIB-148 | 65.16 | 0.47 | 728.43 | 10.30 | 89.10 | 3.89 | 10.16 | 0.10 | 537.20 | 54.05 | 151328.07 | 1916.43 |
| NIND | N | NIB-165 | 40.54 | 1.52 | 62.59 | 2.03 | 24.07 | 4.03 | 42.95 | 4.70 | 28.68 | 15.04 | 32766.98 | 372.75 |
| NIND | N | NIB-206 | 79.31 | 12.74 | 43.06 | 10.31 | 16.52 | 5.93 | 99.22 | 92.95 | 15.92 | 3.71 | 21831.27 | 1249.01 |
| NIND | N | NIB-208 | 42.22 | 4.79 | 45.25 | 5.06 | 11.92 | 3.25 | 51.57 | 12.76 | 22.27 | 11.76 | 13153.99 | 763.83 |
| NIND | N | NIB-212 | 50.16 | 1.58 | 36.08 | 1.41 | 27.16 | 12.24 | 105.11 | 8.88 | 29.16 | 10.26 | 32817.01 | 1368.73 |
| NIND | N | NIB-215 | 58.25 | 3.94 | 54.18 | 3.29 | 19.15 | 3.24 | 22.94 | 2.07 | 27.18 | 1.75 | 23741.53 | 502.37 |
| SPMS | Y | B52 | 31.00 | 24.72 | 71.93 | 2.63 | 8.58 | 5.76 | 2.84 | 3.38 | 8.10 | 1.46 | 20487.09 | 1497.62 |
| SPMS | Y | B64 | 31.04 | 6.11 | 44.66 | 6.65 | 7.62 | 1.50 | 17.36 | 2.58 | 9.15 | 2.09 | 9598.71 | 892.97 |
| SPMS | Y | B80 | 38.27 | 16.88 | 95.52 | 12.11 | 5.81 | 1.76 | 9.70 | 6.78 | 7.79 | 1.07 | 9597.48 | 1115.08 |
| SPMS | Y | B71 | 18.39 | 3.98 | 54.61 | 6.31 | 24.83 | 4.45 | 77.94 | 6.75 | 26.87 | 1.75 | 31341.70 | 3313.60 |
| SPMS | Y | B1_96 | 39.77 | 3.56 | 47.90 | 8.56 | 3.29 | 0.53 | 8.51 | 2.29 | 4.92 | 2.07 | 6046.64 | 304.09 |
| SPMS | Y | B2_96 | 61.38 | 9.05 | 49.56 | 2.27 | 4.05 | 0.63 | 9.21 | 1.03 | 6.58 | 1.67 | 5958.38 | 724.91 |
| SPMS | Y | B1_98 | 22.51 | 7.41 | 45.48 | 9.25 | 7.48 | 5.54 | 20.59 | 19.34 | 9.97 | 6.77 | 9054.06 | 5099.50 |
| SPMS | Y | B2_98 | 31.97 | 5.46 | 42.10 | 3.26 | 9.00 | 0.72 | 25.82 | 8.29 | 11.08 | 2.78 | 9755.75 | 253.23 |
| SPMS | Y | B1_117 | 69.47 | 14.75 | 24.41 | 6.86 | 9.99 | 5.09 | 13.76 | 15.24 | 10.22 | 10.69 | 16676.78 | 7437.56 |
| SPMS | Y | B2_117 | 41.12 | 4.87 | 62.39 | 3.95 | 7.85 | 0.88 | 8.13 | 7.62 | 9.37 | 2.20 | 12602.61 | 1089.22 |
|  |  |  |  |  |  |  |  |  |  |  |  |  |  |  |
| DG | Treat-ment | Sample Code | KLKB1 | Std | NFH | Std | SAMP | Std | 1433F | Std | APOE | Std | NID2 | Std |
| SPMS | N | A4 | 1263.07 | 43.49 | 44.05 | 23.12 | 32.76 | 2.81 | 12.07 | 1.08 | 891.71 | 93.27 | 31.06 | 15.28 |
| SPMS | N | A79 | 890.13 | 29.75 | 35.75 | 3.03 | 25.47 | 2.78 | 7.75 | 1.23 | 1054.37 | 27.72 | 26.57 | 12.51 |
| SPMS | N | A37 | 1806.08 | 63.50 | 66.78 | 6.00 | 42.35 | 1.90 | 15.02 | 1.06 | 601.69 | 36.73 | 26.48 | 11.88 |
| SPMS | N | A78 | 1522.98 | 97.26 | 65.85 | 4.29 | 38.22 | 4.82 | 13.43 | 1.58 | 210.33 | 20.66 | 27.31 | 18.57 |
| SPMS | N | A30 | 1958.55 | 126.04 | 82.62 | 4.69 | 48.87 | 5.17 | 15.83 | 2.70 | 541.04 | 58.18 | 27.53 | 13.64 |
| SPMS | N | A26 | 2439.70 | 284.80 | 112.75 | 11.43 | 60.46 | 10.23 | 23.03 | 4.24 | 722.14 | 62.83 | 42.62 | 34.71 |
| SPMS | N | A1_59 | 1181.80 | 239.91 | 47.53 | 7.32 | 34.34 | 6.37 | 7.81 | 3.57 | 812.34 | 29.25 | 49.33 | 11.40 |
| SPMS | N | A2_59 | 1203.24 | 240.94 | 40.78 | 15.41 | 33.00 | 4.00 | 7.42 | 5.90 | 949.84 | 82.59 | 20.75 | 8.83 |
| SPMS | N | A1_70 | 2003.41 | 62.42 | 86.54 | 4.56 | 57.69 | 6.11 | 19.81 | 1.12 | 542.39 | 35.02 | 34.51 | 30.60 |
| SPMS | N | A2_70 | 1962.21 | 106.95 | 90.79 | 12.54 | 39.42 | 5.60 | 16.22 | 2.39 | 371.03 | 37.29 | 10.18 | 11.96 |
| SPMS | N | A1_62 | 1407.37 | 62.96 | 62.42 | 3.77 | 34.00 | 3.97 | 11.58 | 2.71 | 469.18 | 33.81 | 13.74 | 19.40 |
| SPMS | N | A2_62 | 1573.58 | 129.02 | 72.72 | 15.54 | 43.27 | 5.89 | 9.43 | 4.94 | 540.53 | 78.50 | 17.11 | 18.03 |
| SPMS | N | A1_90 | 1388.49 | 37.95 | 60.04 | 8.06 | 36.59 | 1.00 | 12.15 | 1.89 | 889.68 | 25.89 | 30.01 | 15.80 |
| SPMS | N | A2_90 | 1394.66 | 89.64 | 62.13 | 9.41 | 38.42 | 2.60 | 11.73 | 7.16 | 932.89 | 17.50 | 31.88 | 9.62 |
| SPMS | N | NIB-156 | 3599.50 | 150.51 | 188.68 | 5.07 | 114.94 | 17.78 | 33.66 | 2.43 | 1127.27 | 1.79 | NF |  |
| SPMS | N | NIB-164 | 2909.79 | 85.12 | 136.57 | 9.02 | 109.22 | 17.75 | 20.06 | 0.16 | 1470.43 | 31.42 | NF |  |
| SPMS | N | NIB-169 | 1613.23 | 72.41 | 82.17 | 2.70 | 54.60 | 2.33 | 17.40 | 3.09 | 1277.24 | 27.17 | NF |  |
| SPMS | N | NIB-176 | 1234.77 | 13.28 | 78.98 | 6.78 | 81.04 | 12.63 | 12.58 | 2.01 | 1864.52 | 36.26 | NF |  |
| SPMS | N | NIB-187 | 1019.25 | 24.86 | 113.45 | 15.71 | 66.84 | 8.21 | 79.64 | 8.00 | 5288.30 | 123.75 | NF |  |
| SPMS | N | NIB-188 | 2551.99 | 58.84 | 132.19 | 11.49 | 109.06 | 12.17 | 27.72 | 6.12 | 1788.47 | 10.26 | NF |  |
| SPMS | N | NIB-189 | 4453.34 | 199.72 | 207.59 | 19.58 | 149.63 | 2.94 | 32.48 | 2.15 | 1305.40 | 38.01 | NF |  |
| SPMS | N | NIB-214 | 3286.91 | 494.41 | 161.01 | 1.81 | 105.54 | 14.68 | 21.85 | 3.40 | 1892.70 | 245.39 | NF |  |
| SPMS | N | NIB-077 | 2026.02 | 18.52 | 93.93 | 5.05 | 73.52 | 4.31 | 17.51 | 2.13 | 1654.41 | 123.53 | NF |  |
| SPMS | N | NIB-088 | 1443.71 | 18.31 | 163.83 | 7.29 | 77.97 | 6.71 | 126.12 | 6.38 | 7928.90 | 194.28 | NF |  |
| SPMS | N | NIB-098 | 1758.74 | 32.02 | 77.99 | 0.87 | 69.43 | 10.02 | 11.43 | 2.21 | 1501.80 | 11.95 | NF |  |
| SPMS | N | NIB-107 | 4331.08 | 371.36 | 196.96 | 5.71 | 141.93 | 3.49 | 31.01 | 1.89 | 2455.17 | 51.99 | NF |  |
| HC | N | 60167 | 808.31 | 80.14 | 24.98 | 13.06 | 19.63 | 3.93 | 5.86 | 0.92 | 604.49 | 23.64 | 7.47 | 7.65 |
| HC | N | 80155 | 1261.62 | 48.76 | 44.92 | 2.79 | 30.95 | 4.08 | 7.85 | 0.96 | 809.11 | 69.47 | 8.74 | 10.67 |
| HC | N | 100263 | 1415.27 | 1102.82 | 101.30 | 3.87 | 58.04 | 9.49 | 19.45 | 0.99 | 941.32 | 25.56 | 20.23 | 23.59 |
| HC | N | 100129 | 627.63 | 484.68 | 41.49 | 6.34 | 25.24 | 4.47 | 7.38 | 1.44 | 1313.69 | 114.14 | 15.19 | 8.34 |
| HC | N | 60023 | 143.21 | 3.83 | 95.78 | 7.69 | 51.09 | 7.83 | 14.59 | 0.77 | 879.71 | 17.70 | NF |  |
| HC | N | 60163 | 330.00 | 23.91 | 115.66 | 8.93 | 68.69 | 10.39 | 15.77 | 0.51 | 923.38 | 23.72 | NF |  |
| HC | N | 90092 | 1332.70 | 44.27 | 66.30 | 5.21 | 50.36 | 5.13 | 10.32 | 7.62 | 2105.05 | 24.88 | NF |  |
| HC | N | 90149 | 2363.91 | 3.20 | 97.85 | 9.35 | 68.85 | 13.17 | 17.87 | 1.11 | 1225.85 | 12.32 | NF |  |
| HC | N | 70118 | 2584.08 | 121.25 | 129.97 | 2.72 | 81.98 | 16.40 | 25.24 | 4.40 | 1155.51 | 7.89 | NF |  |
| HC | N | 40010 | 2222.48 | 72.66 | 100.41 | 3.59 | 50.56 | 3.57 | 14.44 | 4.08 | 1336.89 | 25.46 | NF |  |
| NIND | N | NIB-091 | 541.39 | 56.55 | 66.72 | 14.90 | 35.60 | 2.47 | 60.26 | 6.56 | 3832.37 | 533.16 | NF |  |
| NIND | N | NIB-103 | 1597.14 | 21.22 | 101.69 | 5.89 | 54.14 | 2.80 | 10.64 | 5.72 | 1742.93 | 33.37 | NF |  |
| NIND | N | NIB-114 | 809.78 | 2.72 | 95.74 | 8.66 | 63.08 | 9.12 | 64.48 | 9.40 | 6379.58 | 265.18 | NF |  |
| NIND | N | NIB-134 | 1120.73 | 25.22 | 70.87 | 10.36 | 55.77 | 4.08 | 9.79 | 4.90 | 2328.28 | 158.34 | NF |  |
| NIND | N | NIB-137 | 691.09 | 13.65 | 78.79 | 6.25 | 46.08 | 3.51 | 62.72 | 4.81 | 8999.25 | 21.29 | NF |  |
| NIND | N | NIB-144 | 3062.75 | 63.81 | 86.07 | 68.51 | 93.31 | 10.05 | 21.72 | 2.17 | 3480.06 | 69.45 | NF |  |
| NIND | N | NIB-148 | 777.99 | 27.83 | 96.09 | 4.28 | 57.46 | 8.19 | 68.48 | 19.02 | 6432.46 | 174.87 | NF |  |
| NIND | N | NIB-165 | 2116.35 | 5.37 | 108.86 | 17.21 | 79.05 | 6.42 | 21.39 | 2.63 | 1806.73 | 43.44 | NF |  |
| NIND | N | NIB-206 | 1405.67 | 19.99 | 69.27 | 8.00 | 51.78 | 5.23 | 8.39 | 4.45 | 1356.88 | 86.09 | NF |  |
| NIND | N | NIB-208 | 1180.16 | 82.93 | 62.88 | 14.08 | 56.82 | 11.47 | 8.14 | 2.14 | 1511.77 | 164.34 | NF |  |
| NIND | N | NIB-212 | 3136.29 | 96.87 | 150.26 | 5.10 | 111.38 | 15.21 | 20.85 | 3.75 | 1267.63 | 151.06 | NF |  |
| NIND | N | NIB-215 | 1618.7 | 48.5 | 83.40 | 7.00 | 50.79 | 5.73 | 12.79 | 1.42 | 1539.638 | 33.1403 | NF |  |
| SPMS | Y | B52 | 1218.31 | 73.15 | 22.57 | 6.05 | 20.17 | 5.88 | 3.87 | 5.00 | 1385.99 | 89.36 | 13.55 | 11.87 |
| SPMS | Y | B64 | 895.69 | 80.51 | 38.53 | 3.01 | 24.10 | 2.69 | 5.22 | 5.40 | 644.75 | 56.76 | 18.12 | 13.95 |
| SPMS | Y | B80 | 933.49 | 86.79 | 37.30 | 9.26 | 22.12 | 8.21 | 4.99 | 4.16 | 1644.59 | 173.72 | 29.51 | 17.15 |
| SPMS | Y | B71 | 2823.48 | 122.02 | 117.43 | 11.47 | 61.24 | 8.29 | 16.38 | 8.83 | 691.25 | 69.14 | 27.16 | 17.34 |
| SPMS | Y | B1_96 | 563.29 | 10.43 | 20.80 | 4.21 | 16.16 | 3.52 | 3.23 | 1.56 | 686.49 | 39.62 | 22.68 | 7.30 |
| SPMS | Y | B2_96 | 592.83 | 22.59 | 21.64 | 1.69 | 20.95 | 1.62 | 4.98 | 2.26 | 703.82 | 8.99 | 23.61 | 8.41 |
| SPMS | Y | B1_98 | 236.19 | 202.07 | 45.38 | 29.19 | 23.03 | 13.03 | 7.47 | 4.91 | 625.97 | 162.36 | 25.36 | 18.52 |
| SPMS | Y | B2_98 | 481.73 | 489.16 | 41.05 | 4.22 | 26.95 | 3.99 | 4.74 | 3.47 | 647.64 | 52.16 | 20.73 | 8.59 |
| SPMS | Y | B1_117 | 254.71 | 9.41 | 65.40 | 22.85 | 28.77 | 25.78 | 11.35 | 3.82 | 892.75 | 544.73 | 22.49 | 30.61 |
| SPMS | Y | B2_117 | 1294.48 | 110.84 | 39.43 | 8.42 | 24.83 | 4.78 | 7.02 | 1.24 | 743.42 | 48.16 | 11.05 | 5.77 |
|  |  |  |  |  |  |  |  |  |  |  |  |  |  |  |
| DG | Treat-ment | Sample Code | PEDF | Std | TAU | Std | KLK6 | Std | AMD | Std | VGF | Std | A2MG | Std |
| SPMS | N | A4 | 450.33 | 11.12 | 35.45 | 2.82 | 578.88 | 26.72 | 11.35 | 1.28 | 248.78 | 10.40 | 439.41 | 61.09 |
| SPMS | N | A79 | 529.71 | 25.49 | 25.08 | 2.11 | 907.65 | 62.03 | 19.85 | 3.05 | 277.51 | 17.17 | 324.74 | 24.22 |
| SPMS | N | A37 | 431.39 | 17.45 | 43.67 | 5.20 | 691.70 | 53.06 | 20.29 | 2.88 | 141.61 | 9.69 | 256.42 | 6.32 |
| SPMS | N | A78 | 436.56 | 40.76 | 40.00 | 8.00 | 371.11 | 29.11 | 16.42 | 4.19 | 66.39 | 3.22 | 191.16 | 17.69 |
| SPMS | N | A30 | 535.55 | 58.36 | 50.20 | 7.79 | 899.68 | 115.40 | 17.55 | 5.21 | 223.92 | 20.08 | 318.42 | 19.09 |
| SPMS | N | A26 | 532.53 | 64.39 | 66.00 | 7.28 | 512.22 | 62.42 | 36.38 | 23.71 | 104.40 | 8.85 | 337.56 | 34.65 |
| SPMS | N | A1_59 | 600.72 | 15.93 | 31.89 | 4.21 | 737.21 | 59.35 | 29.13 | 19.72 | 418.56 | 10.97 | 378.91 | 28.45 |
| SPMS | N | A2_59 | 639.61 | 59.72 | 31.92 | 4.32 | 913.45 | 163.32 | 29.46 | 7.69 | 520.83 | 54.61 | 361.24 | 53.41 |
| SPMS | N | A1_70 | 488.78 | 19.51 | 65.57 | 9.15 | 449.11 | 7.96 | 32.28 | 11.11 | 144.52 | 7.37 | 315.24 | 8.35 |
| SPMS | N | A2_70 | 390.40 | 53.36 | 44.88 | 6.19 | 374.19 | 35.68 | 20.40 | 2.09 | 94.13 | 7.36 | 251.05 | 23.95 |
| SPMS | N | A1_62 | 386.38 | 53.47 | 34.98 | 6.92 | 487.62 | 65.99 | 51.08 | 13.02 | 71.84 | 4.80 | 187.92 | 8.15 |
| SPMS | N | A2_62 | 429.68 | 62.21 | 40.24 | 9.12 | 501.78 | 59.81 | 24.72 | 3.92 | 76.50 | 9.59 | 230.89 | 58.01 |
| SPMS | N | A1_90 | 493.94 | 17.10 | 37.04 | 4.15 | 712.07 | 47.84 | 26.13 | 2.91 | 272.86 | 11.41 | 134.83 | 17.77 |
| SPMS | N | A2_90 | 525.19 | 35.55 | 40.79 | 3.84 | 761.83 | 49.97 | 53.52 | 35.68 | 237.13 | 4.97 | 154.08 | 16.02 |
| SPMS | N | NIB-156 | 887.12 | 37.15 | 128.29 | 19.92 | 945.89 | 38.02 | 31.23 | 27.90 | 201.00 | 3.75 | 372.75 | 20.31 |
| SPMS | N | NIB-164 | 777.82 | 21.70 | 125.69 | 6.49 | 1537.27 | 21.90 | 30.45 | 2.46 | 358.68 | 32.30 | 558.01 | 27.62 |
| SPMS | N | NIB-169 | 870.29 | 21.90 | 55.73 | 4.03 | 716.36 | 26.59 | 11.36 | 3.91 | 131.62 | 4.79 | 297.09 | 6.88 |
| SPMS | N | NIB-176 | 836.07 | 25.07 | 86.71 | 3.77 | 836.03 | 25.26 | 31.13 | 3.11 | 233.24 | 12.67 | 266.29 | 8.31 |
| SPMS | N | NIB-187 | 711.35 | 46.24 | 80.09 | 7.56 | 771.23 | 9.13 | 855.95 | 44.00 | 119.45 | 3.41 | 803.98 | 23.53 |
| SPMS | N | NIB-188 | 1079.78 | 12.71 | 123.60 | 12.64 | 1686.25 | 95.52 | 16.06 | 2.08 | 407.68 | 19.21 | 403.23 | 25.19 |
| SPMS | N | NIB-189 | 854.34 | 43.31 | 156.55 | 14.86 | 1052.44 | 29.93 | 29.16 | 2.61 | 176.45 | 9.11 | 350.02 | 23.41 |
| SPMS | N | NIB-214 | 1193.68 | 166.99 | 112.49 | 18.72 | 1352.29 | 163.80 | 44.82 | 6.76 | 322.17 | 39.82 | 421.58 | 32.18 |
| SPMS | N | NIB-077 | 758.41 | 48.42 | 73.97 | 4.47 | 1256.31 | 22.47 | 18.02 | 1.32 | 422.54 | 7.56 | 358.62 | 12.18 |
| SPMS | N | NIB-088 | 1149.95 | 21.81 | 84.49 | 7.83 | 1438.37 | 39.93 | 1084.30 | 16.48 | 285.61 | 12.83 | 1294.18 | 15.67 |
| SPMS | N | NIB-098 | 750.78 | 17.67 | 67.54 | 7.77 | 1508.81 | 47.21 | 10.61 | 1.88 | 428.31 | 6.58 | 360.90 | 12.47 |
| SPMS | N | NIB-107 | 1212.71 | 27.55 | 138.23 | 16.41 | 2200.37 | 37.87 | 73.28 | 7.28 | 664.68 | 52.62 | 545.93 | 18.47 |
| HC | N | 60167 | 233.30 | 14.11 | 19.10 | 4.21 | 524.87 | 18.87 | 27.77 | 16.63 | 131.69 | 6.35 | 190.97 | 9.63 |
| HC | N | 80155 | 541.59 | 59.82 | 27.38 | 2.92 | 702.18 | 89.85 | 15.75 | 7.01 | 219.13 | 22.34 | 252.90 | 20.00 |
| HC | N | 100263 | 486.60 | 19.90 | 54.82 | 7.94 | 702.29 | 43.79 | 30.01 | 5.82 | 159.05 | 14.89 | 300.10 | 15.40 |
| HC | N | 100129 | 586.61 | 36.88 | 23.70 | 7.08 | 922.56 | 26.15 | 11.88 | 4.51 | 488.59 | 36.46 | 370.33 | 31.53 |
| HC | N | 60023 | 509.41 | 16.45 | 59.03 | 3.36 | 821.31 | 5.89 | 57.85 | 26.27 | 221.48 | 16.33 | 206.27 | 7.22 |
| HC | N | 60163 | 793.26 | 6.21 | 66.80 | 7.33 | 1083.58 | 37.70 | 79.41 | 45.40 | 199.46 | 7.80 | 254.08 | 9.09 |
| HC | N | 90092 | 796.93 | 39.02 | 46.81 | 6.38 | 1753.46 | 47.73 | 22.88 | 0.76 | 646.38 | 8.42 | 194.92 | 6.41 |
| HC | N | 90149 | 578.29 | 19.77 | 69.40 | 4.17 | 970.66 | 92.13 | 21.20 | 2.89 | 308.37 | 13.50 | 367.29 | 8.73 |
| HC | N | 70118 | 599.12 | 29.61 | 79.24 | 9.46 | 672.50 | 32.52 | 18.08 | 5.85 | 215.52 | 11.04 | 292.33 | 5.77 |
| HC | N | 40010 | 655.35 | 18.02 | 52.10 | 9.27 | 1052.00 | 38.27 | 13.14 | 6.77 | 257.08 | 11.88 | 350.89 | 8.81 |
| NIND | N | NIB-091 | 672.91 | 132.51 | 41.45 | 11.41 | 1177.64 | 219.98 | 576.54 | 35.44 | 158.56 | 20.13 | 940.51 | 202.34 |
| NIND | N | NIB-103 | 1239.86 | 31.78 | 61.04 | 13.85 | 1685.60 | 68.97 | 7.36 | 13.04 | 302.30 | 12.18 | 416.87 | 9.37 |
| NIND | N | NIB-114 | 914.33 | 37.69 | 69.54 | 2.38 | 1442.51 | 90.06 | 784.44 | 24.91 | 277.54 | 6.81 | 879.70 | 14.74 |
| NIND | N | NIB-134 | 777.40 | 58.28 | 51.22 | 2.42 | 2009.96 | 9.67 | 33.39 | 4.42 | 606.91 | 20.23 | 482.57 | 2.89 |
| NIND | N | NIB-137 | 938.88 | 43.23 | 53.55 | 1.69 | 2135.99 | 14.58 | 678.20 | 34.51 | 511.91 | 14.22 | 1073.84 | 97.86 |
| NIND | N | NIB-144 | 1230.48 | 29.87 | 89.91 | 12.95 | 2195.75 | 17.58 | 24.80 | 10.04 | 1014.01 | 47.97 | 638.67 | 4.90 |
| NIND | N | NIB-148 | 952.03 | 14.88 | 70.12 | 6.02 | 1477.00 | 25.96 | 784.55 | 16.51 | 305.55 | 13.20 | 1102.17 | 18.90 |
| NIND | N | NIB-165 | 886.72 | 14.93 | 90.25 | 11.74 | 1556.65 | 112.75 | 14.17 | 4.09 | 444.82 | 11.55 | 346.03 | 9.49 |
| NIND | N | NIB-206 | 787.66 | 10.89 | 54.31 | 0.65 | 1043.15 | 43.48 | 7.03 | 1.29 | 265.22 | 10.56 | 297.05 | 10.99 |
| NIND | N | NIB-208 | 638.83 | 15.97 | 49.62 | 5.52 | 1249.94 | 105.47 | 19.28 | 3.33 | 579.74 | 21.15 | 287.76 | 24.91 |
| NIND | N | NIB-212 | 699.78 | 21.53 | 143.15 | 7.90 | 1014.69 | 11.54 | 19.94 | 1.07 | 307.32 | 6.21 | 277.97 | 10.84 |
| NIND | N | NIB-215 | 916.66 | 23.50 | 51.32 | 3.65 | 1441.27 | 48.67 | 12.347 | 6.408 | 454.09 | 5.79 | 380.72 | 7.91 |
| SPMS | Y | B52 | 658.67 | 298.88 | 17.49 | 18.76 | 1374.86 | 82.36 | 12.69 | 7.19 | 437.89 | 32.66 | 348.12 | 165.58 |
| SPMS | Y | B64 | 313.70 | 38.14 | 24.06 | 2.62 | 546.06 | 66.34 | 23.79 | 4.30 | 145.16 | 7.93 | 206.70 | 13.85 |
| SPMS | Y | B80 | 617.19 | 33.65 | 21.54 | 6.25 | 1004.47 | 46.81 | 20.08 | 7.67 | 346.28 | 33.58 | 361.62 | 40.19 |
| SPMS | Y | B71 | 357.91 | 33.16 | 67.14 | 9.65 | 444.33 | 26.58 | 39.13 | 6.74 | 89.20 | 5.44 | 259.62 | 15.45 |
| SPMS | Y | B1_96 | 518.74 | 29.55 | 15.41 | 3.11 | 752.44 | 44.72 | 26.57 | 15.57 | 189.66 | 8.17 | 198.21 | 9.95 |
| SPMS | Y | B2_96 | 581.68 | 41.81 | 18.61 | 2.74 | 805.12 | 33.06 | 40.33 | 7.86 | 192.87 | 7.60 | 212.59 | 7.50 |
| SPMS | Y | B1_98 | 452.44 | 29.51 | 24.90 | 15.81 | 699.42 | 90.78 | 23.22 | 2.81 | 135.74 | 21.76 | 201.95 | 74.15 |
| SPMS | Y | B2_98 | 354.41 | 18.65 | 28.84 | 7.03 | 663.49 | 45.68 | 23.11 | 12.07 | 155.34 | 7.30 | 199.16 | 12.60 |
| SPMS | Y | B1_117 | 604.37 | 15.22 | 24.70 | 25.36 | 1029.96 | 551.27 | 17.19 | 16.31 | 286.95 | 116.27 | 244.56 | 186.68 |
| SPMS | Y | B2_117 | 639.01 | 23.63 | 23.47 | 3.64 | 823.72 | 28.69 | 14.69 | 3.58 | 209.65 | 9.45 | 213.50 | 14.29 |
|  |  |  |  |  |  |  |  |  |  |  |  |  |  |  |
| DG | Treat-ment | Sample Code | AGRIN | Std | A4 | Std | SODC | Std |  |  |  |  |  |  |
| SPMS | N | A4 | 1785.58 | 22.64 | 109.04 | 11.60 | 101.59 | 7.15 |  |  |  |  |  |  |
| SPMS | N | A79 | 1325.07 | 51.11 | 177.80 | 4.77 | 125.51 | 9.60 |  |  |  |  |  |  |
| SPMS | N | A37 | 2527.75 | 67.99 | 60.36 | 18.07 | 75.22 | 6.18 |  |  |  |  |  |  |
| SPMS | N | A78 | 2205.28 | 85.58 | 45.23 | 5.98 | 37.95 | 3.92 |  |  |  |  |  |  |
| SPMS | N | A30 | 2855.23 | 158.79 | 106.86 | 4.76 | 84.07 | 11.40 |  |  |  |  |  |  |
| SPMS | N | A26 | 3579.60 | 412.37 | 98.83 | 20.36 | 63.48 | 8.65 |  |  |  |  |  |  |
| SPMS | N | A1_59 | 1741.73 | 346.83 | 89.00 | 31.79 | 114.47 | 9.20 |  |  |  |  |  |  |
| SPMS | N | A2_59 | 1691.68 | 348.77 | 84.50 | 63.01 | 146.05 | 33.91 |  |  |  |  |  |  |
| SPMS | N | A1_70 | 3017.84 | 80.96 | 21.39 | 4.35 | 66.32 | 1.95 |  |  |  |  |  |  |
| SPMS | N | A2_70 | 2853.32 | 137.70 | 42.49 | 3.05 | 55.45 | 8.77 |  |  |  |  |  |  |
| SPMS | N | A1_62 | 1995.76 | 61.44 | 70.92 | 6.94 | 47.90 | 5.43 |  |  |  |  |  |  |
| SPMS | N | A2_62 | 2298.35 | 279.30 | 83.20 | 4.54 | 53.86 | 8.96 |  |  |  |  |  |  |
| SPMS | N | A1_90 | 2054.51 | 45.36 | 87.11 | 11.13 | 97.81 | 2.39 |  |  |  |  |  |  |
| SPMS | N | A2_90 | 2044.86 | 108.61 | 77.65 | 27.05 | 102.65 | 5.11 |  |  |  |  |  |  |
| SPMS | N | NIB-156 | 5494.50 | 111.17 | 104.43 | 15.98 | 47.97 | 3.73 |  |  |  |  |  |  |
| SPMS | N | NIB-164 | 4305.08 | 74.45 | 167.56 | 9.88 | 90.79 | 1.66 |  |  |  |  |  |  |
| SPMS | N | NIB-169 | 2247.22 | 53.77 | 77.24 | 0.17 | 54.50 | 1.65 |  |  |  |  |  |  |
| SPMS | N | NIB-176 | 1692.73 | 23.37 | 130.78 | 7.36 | 69.94 | 1.39 |  |  |  |  |  |  |
| SPMS | N | NIB-187 | 1475.73 | 126.97 | 94.99 | 3.67 | 102.64 | 1.91 |  |  |  |  |  |  |
| SPMS | N | NIB-188 | 3723.92 | 35.55 | 236.86 | 15.06 | 101.59 | 8.54 |  |  |  |  |  |  |
| SPMS | N | NIB-189 | 6529.23 | 224.03 | 147.90 | 2.58 | 68.67 | 4.54 |  |  |  |  |  |  |
| SPMS | N | NIB-214 | 4917.15 | 629.16 | 153.50 | 20.97 | 102.31 | 15.81 |  |  |  |  |  |  |
| SPMS | N | NIB-077 | 2917.84 | 31.31 | 167.24 | 7.87 | 73.41 | 2.61 |  |  |  |  |  |  |
| SPMS | N | NIB-088 | 2188.25 | 44.58 | 150.17 | 3.57 | 155.04 | 0.97 |  |  |  |  |  |  |
| SPMS | N | NIB-098 | 2440.06 | 81.32 | 254.15 | 12.71 | 71.95 | 2.63 |  |  |  |  |  |  |
| SPMS | N | NIB-107 | 6207.78 | 957.42 | 292.73 | 14.60 | 146.51 | 7.83 |  |  |  |  |  |  |
| HC | N | 60167 | 1154.62 | 109.16 | 85.68 | 8.90 | 68.01 | 5.73 |  |  |  |  |  |  |
| HC | N | 80155 | 1818.87 | 39.00 | 98.26 | 47.01 | 94.49 | 7.34 |  |  |  |  |  |  |
| HC | N | 100263 | 1905.61 | 1437.19 | 87.86 | 8.08 | 81.77 | 15.62 |  |  |  |  |  |  |
| HC | N | 100129 | 904.19 | 699.94 | 214.40 | 15.30 | 118.70 | 51.62 |  |  |  |  |  |  |
| HC | N | 60023 | 208.35 | 3.60 | 141.43 | 10.06 | 28.39 | 0.64 |  |  |  |  |  |  |
| HC | N | 60163 | 484.06 | 12.73 | 145.47 | 3.57 | 46.29 | 1.71 |  |  |  |  |  |  |
| HC | N | 90092 | 1886.53 | 84.01 | 267.65 | 10.09 | 116.53 | 4.56 |  |  |  |  |  |  |
| HC | N | 90149 | 3229.61 | 58.81 | 129.08 | 3.99 | 72.10 | 0.95 |  |  |  |  |  |  |
| HC | N | 70118 | 3636.24 | 131.24 | 107.48 | 7.79 | 56.58 | 3.08 |  |  |  |  |  |  |
| HC | N | 40010 | 3099.50 | 61.79 | 117.29 | 8.63 | 79.09 | 2.16 |  |  |  |  |  |  |
| NIND | N | NIB-091 | 835.19 | 97.39 | 88.01 | 9.87 | 140.47 | 13.73 |  |  |  |  |  |  |
| NIND | N | NIB-103 | 2182.71 | 78.37 | 194.65 | 9.85 | 91.35 | 0.08 |  |  |  |  |  |  |
| NIND | N | NIB-114 | 1280.07 | 37.22 | 193.07 | 7.65 | 137.11 | 5.19 |  |  |  |  |  |  |
| NIND | N | NIB-134 | 1664.55 | 46.91 | 231.69 | 1.36 | 132.38 | 3.97 |  |  |  |  |  |  |
| NIND | N | NIB-137 | 1075.45 | 17.85 | 267.42 | 9.43 | 192.23 | 11.18 |  |  |  |  |  |  |
| NIND | N | NIB-144 | 4213.39 | 31.56 | 448.70 | 6.16 | 154.27 | 7.97 |  |  |  |  |  |  |
| NIND | N | NIB-148 | 1175.31 | 23.58 | 134.60 | 9.72 | 124.02 | 7.51 |  |  |  |  |  |  |
| NIND | N | NIB-165 | 2943.48 | 43.99 | 239.77 | 1.09 | 87.30 | 2.31 |  |  |  |  |  |  |
| NIND | N | NIB-206 | 2004.38 | 49.03 | 123.35 | 10.33 | 69.68 | 2.12 |  |  |  |  |  |  |
| NIND | N | NIB-208 | 1676.52 | 71.88 | 266.66 | 23.09 | 83.02 | 11.35 |  |  |  |  |  |  |
| NIND | N | NIB-212 | 4446.68 | 192.72 | 189.66 | 8.16 | 66.97 | 5.35 |  |  |  |  |  |  |
| NIND | N | NIB-215 | 2267.874 | 26.52559 | 201.28 | 5.93 | 85.303 | 1.477 |  |  |  |  |  |  |
| SPMS | Y | B52 | 1492.00 | 960.15 | 21.72 | 23.73 | 141.73 | 6.45 |  |  |  |  |  |  |
| SPMS | Y | B64 | 1298.95 | 106.90 | 97.92 | 8.11 | 83.64 | 9.90 |  |  |  |  |  |  |
| SPMS | Y | B80 | 1364.23 | 174.23 | 129.75 | 75.41 | 165.10 | 25.15 |  |  |  |  |  |  |
| SPMS | Y | B71 | 4001.59 | 204.96 | 64.33 | 5.78 | 73.26 | 6.67 |  |  |  |  |  |  |
| SPMS | Y | B1_96 | 802.23 | 18.99 | 103.49 | 12.64 | 80.40 | 6.16 |  |  |  |  |  |  |
| SPMS | Y | B2_96 | 881.57 | 22.33 | 108.59 | 10.79 | 87.57 | 2.69 |  |  |  |  |  |  |
| SPMS | Y | B1_98 | 336.47 | 281.35 | 108.05 | 16.60 | 50.34 | 18.61 |  |  |  |  |  |  |
| SPMS | Y | B2_98 | 750.85 | 759.09 | 127.29 | 15.33 | 64.04 | 21.30 |  |  |  |  |  |  |
| SPMS | Y | B1_117 | 1865.95 | 2008.27 | 113.26 | 109.49 | 128.12 | 65.57 |  |  |  |  |  |  |
| SPMS | Y | B2_117 | 1825.20 | 211.70 | 70.27 | 49.78 | 101.72 | 2.71 |  |  |  |  |  |  |
